# Cognitive and non-cognitive factors predict pigs’ positions in an aggression social network

**DOI:** 10.1038/s41598-025-02023-w

**Published:** 2025-05-20

**Authors:** Lucy Oldham, Saif Agha, Gareth Arnott, Mark Brims, Irene Camerlink, Agnieszka Futro, Victoria E. Lee, Andrea Doeschl-Wilson, Simon P. Turner

**Affiliations:** 1 Animal Behaviour and Welfare, Animal and Veterinary Sciences Department, Scotland’s Rural College (SRUC), West Mains Rd, Edinburgh, EH9 3 JG UK; 2 The Roslin Institute and R(D)SVS, University of Edinburgh, Easter Bush, Edinburgh, EH25 9RG UK; 3 Institute for Global Food Security, School of Biological Sciences, Queen’s University Belfast, Belfast, BT9 7BL UK; 4 Institute of Genetics and Animal Biotechnology, Polish Academy of Sciences, Ul. Postepu 36 A, Jastrzebiec, 05-552 Magdalenka, Poland

**Keywords:** Cognitive neuroscience, Ecology, Animal behaviour

## Abstract

Social network analysis (SNA) provides a means of understanding animals’ agonistic behaviour in a group. The aim of this study was to use SNA to characterise how individual cognitive performance affects agonistic behaviour. Using 175 pigs, we hypothesised that their choice of opponents would be affected by their ability to discriminate spatial information and to adapt their behaviour when cues were reversed. A spatial discrimination test was conducted; left and right locations were assigned as positive (food reward) and negative (fan) and each pig’s learning speed was recorded. The cues were then reversed, and we tested whether pigs adjusted their behaviour. At age 14 weeks, pigs were regrouped into 14 groups, and their behaviour recorded for 5h, from which weighted and unweighted networks were constructed. Skin lesions were counted after 24h, 1 week and 2 weeks. Males delivered more aggression, and heavier pigs were involved in more aggression. Betweenness centrality (a network position linking otherwise unconnected individuals) increased with network size and decreased with body weight. Passing the reversal learning test predicted more involvement in unilateral aggression. The current study therefore shows links between cognitive performance and aggression and advances the understanding of social network analysis in the context of animal welfare.

## Introduction

Living in dynamic social groups requires an ability to appropriately respond to the behaviour of others and is hypothesised to be cognitively taxing^1–3^. The cognitive component of aggression has not been defined experimentally but navigating agonistic encounters is thought to involve reference memory of previous events and opponents, comparison against current circumstances and selection of appropriate behaviours^4,5^. Pigs are an ideal species to study the effects of cognitive ability on resolution of aggressive contests. Pigs engage in cognitive tasks, are particularly suited to foraging tasks^2^, can distinguish between familiar and unfamiliar conspecifics (whether or not they are genetically related)^6^ and fight to establish a dominance hierarchy under commercial farm conditions. Pig producers commonly perform social mixing of unfamiliar pigs^7^ and group composition is determined by management requirements. Social mixing often occurs several times in a lifetime for growing pigs and breeding sows, each time provoking aggression, which compromises health, welfare and growth^8,9^. Aggression after regrouping is drastically reduced after 24–48 hours^10^, but dominance relationships may be reinforced with brief or non-damaging agonistic behaviour. However, instability in dominance hierarchies can lead to overt fighting even 3 weeks after group mixing^11^.

Cognitive ability could reduce the welfare costs of sudden changes in the environment, in particular the need to navigate new social groups, to form preferential associations with benign or subordinate conspecifics and avoid unresolved conflicts. Stable social relationships with the mother and littermates are disrupted due to abrupt, early weaning and group mixing, so piglets are unable to gradually assimilate new individuals and experiences as wild pigs would^10^, hence the cognitive challenge of broadening the social group beyond immediate relatives is increased in the domestic setting.

We apply cognitive tests to isolate cognitive from non-cognitive factors affecting agonistic behaviour, such as body weight and sex. Although validated tests of cognition in pigs are mostly non-social^2^, we hypothesise that these cognitive skills may generalise across domains, i.e., the ability to categorise opponents using visual information may influence agonistic behaviour. Approximating a rival’s fighting ability using visual information would allow pigs to avoid the costs of fighting^12^. Consistent with this idea of cognitive rival assessment, pairs of pigs which had visual, olfactory and acoustic contact prior to dyadic contests had shorter contests than those which had no contact, including a shorter biting phase^13^.

Social network analysis can be applied to quantify complexity in post-mixing aggression in newly weaned and fattening pigs^14^ and to predict the likelihood of pigs receiving skin lesions. The agonistic behaviour of a pig is affected by the agonistic behaviour of its group mates^15,16^ and by each pig’s previous experience, which affects injury, fatigue, HPA activation^17^ and affective state^18^. Furthermore, social network centrality measures can provide novel insights into the social structure within a pen. For instance, win networks, which distinguish between winners and losers, offer deeper insights into dominance hierarchies, especially when analysing centrality measures such as eigenvector and degree centrality, both of which correlate with pigs’ dominance ranks^19^. Additionally, in competitive environments, pigs may benefit from selecting opponents with higher eigenvector centrality, i.e. animals that have a strong connection to other influential group members^20^, reflecting their prior fighting experience. Fighting against this kind of animal may lead to more efficient contest resolution and reduce the need for prolonged conflicts. We evaluate the social network characteristics of degree, betweenness and eigenvector centrality (defined in Table 1). These metrics have been shown to be suitable for predicting skin lesions received during aggression^14,21^, even in modest group sizes (12–15) of indoor housed pigs^14^. Using this approach, we can generate multiple groups of pigs with known cognitive performance and analyse network traits that are relevant to welfare.

**Table 1:** Definitions of social network traits, adapted from farine and whitehead(2015)^22^.

Term	Definition	Practical implications
Degree centrality *(also “Sociality”, “all-degree centrality”, “unweighted degree centrality”)*	Number of nodes with which the subject directly interacted either as actor or receiver	A pig with high degree centrality interacts aggressively with a high proportion of potential opponents
Weighted degree centrality *(also “strength”)*	Total frequency or duration of interactions between the subject and its directly connected nodes	A pig with high weighted degree centrality interacts aggressively with a high proportion of potential opponents and for a long duration
Outdegree centrality *(weighted outdegree centrality* = *“out-strength”*	Number of directed edges, where the node concerned is the actor. Outdegree centrality can be weighted or unweighted	A pig with high outdegree centrality directs aggression to a high proportion of potential opponents If weighted, the duration of aggression is also high
Indegree centrality *(also “prestige”)* *(weighted indegree centrality* = *“in-strength”)*	Number of directed edges, where the node concerned is the receiver. Indegree centrality can be weighted or unweighted	A pig with high indegree centrality receives aggression from a high proportion of potential opponents If weighted, the duration of aggression is also high
Geodesic distance	Distance in terms of the number of edges which separate nodes A and B, rather than physical distance. This is also called “path length”	If pigs A and B interact aggressively, as do pigs B and C, A and C are connected in a network with a path length of two. Pigs connected with a shorter geodesic distance are most likely to be influenced by each other’s behaviour than those further away
Eigenvector centrality	A node’s influence within the whole network, proportional to the degree of all the nodes that are connected to it. Eigenvector considers not only the number of directly connected nodes, but how well those nodes are connected with other nodes in the network	A pig with high eigenvector centrality is involved in mutual fights with a decided outcome with opponents that themselves have been involved in a high number of mutual fights with a decided outcome, referred to as “experienced” opponents
Betweenness centrality *(also “bridging centrality”)*	The extent to which an individual lies on the shortest geodesic paths between other individuals of a group) The betweenness centrality of a node (x) is found by firstly calculating the ratio of all of the possible paths between two nodes, a and b which run through x. This ratio is calculated for every pair of nodes in the network, and the sum of these ratios is the betweenness centrality of node x. Where several nodes are equally present on the shortest path between two nodes, the betweenness value is divided between these nodes. Weighted betweenness measures take into account the weight of these edges, which reflects the lower cost of travelling through highly weighted indirect connections	A pig with high betweenness exchanges aggression with pigs that are unconnected (have not interacted aggressively with each other), and may be important to the propagation of aggression

We hypothesise that there will be individual differences in performance in cognitive tests, and that this will have consequences for aggression, affecting pigs’ ability to target aggression, win fights and avoid repeated fights.

Hypothesis 1: Cognitive tests reflect individual differences in associative learning speed which is likely to affect how well pigs can discriminate between opponents with higher or lower resource holding potential. Therefore, Hypothesis 1 (a) predicts that pigs with better performance in cognitive tests will engage in fights they will win, reflected in a **higher outdegree centrality and lower indegree centrality** in networks directed by **fight outcome** (more wins and fewer losses).

Pigs may also discriminate on behavioural or physical traits indicating fighting experience. Fighting against pigs that themselves have fighting experience with a variety of opponents may reduce the costs of aggression, since such pigs have gained information on their own RHP and will be more accurate in their decisions. Therefore, Hypothesis 1 (b) predicts that pigs with better performance in cognitive tests will be less likely to exchange agonistic behaviour with pigs that were themselves unconnected (**lower betweenness centrality**) and more likely to fight pigs that were well connected (**higher eigenvector centrality**).

Hypothesis 2: Associative learning tests reflect individual differences in working and reference memory. Remembering individual pigs and the results of agonistic interactions will avoid repeated or prolonged aggressive interactions. Therefore, pigs with better performance in cognitive tests will have **lower weighted degree centrality** (perform and receive less aggression) and **accrue fewer skin lesions** 24 h, 1 week and 2 weeks post-mixing.

Hypothesis 3: Demonstrating an ability to change behaviour according to changes in associations (reversal learning) will result in pigs de-escalating or retreating from fights they will ultimately lose. Therefore, pigs with better performance in cognitive tests will have **lower weighted degree centrality** (receive less aggression) and **accrue fewer skin lesions** 24 h post-mixing.

## Methods

### Ethical note

This study was conducted under licence from the UK Home Office (PPL: P3850 A80D/PP1403242) and with ethical approval from the SRUC Animal Ethics Committee (application number AE 24–2020). Refinements to the barren housing treatment and cognitive tests were made during the first two batches, to minimise stress. All experiments were performed in accordance with relevant guidelines and regulations: ASPA Act 1986; Amendment Regulations 2012 (SI 2012/3039) and are reported following the ARRIVE guidelines.

### Experiment summary

**Fig. 1:**
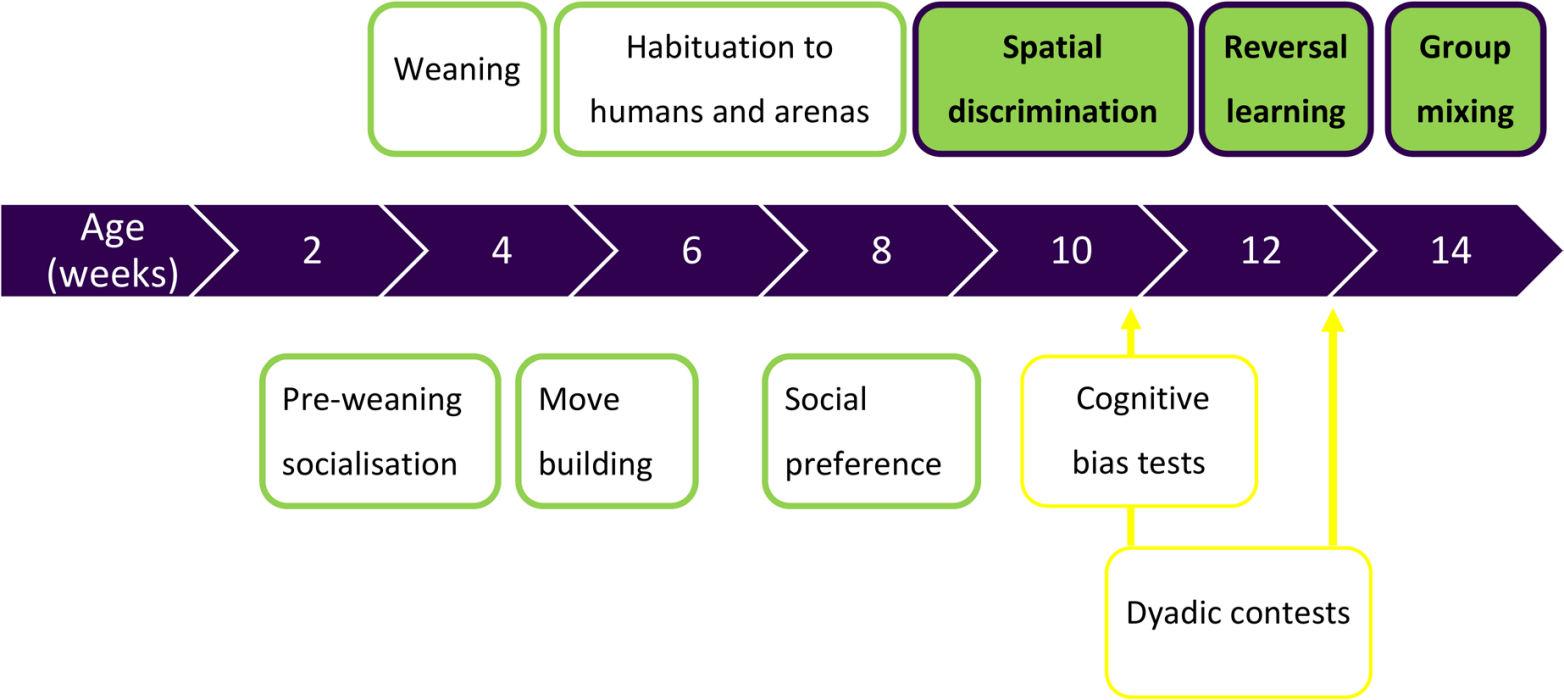
Summary of the experimental timeline. Procedures outlined in yellow were performed as part of a larger study and the results are not included in this paper.

Data were collected from 175 Large White x Landrace x Duroc pigs. Four siblings per litter underwent a spatial discrimination learning task at 10 weeks and a reversal learning task at 12 weeks of age. Full methods of the habituation and training for these tasks are published^23^ (see Fig. 1). At 14 weeks of age, pigs were regrouped into mixed pens of females and intact (uncastrated) males, comprising 14 pigs on average.

### Pig husbandry

Farrowing occurred in free-farrowing pens (4.5 × 2.2 m) and the sow was removed 4 weeks post-farrowing. Pre-weaning socialisation (age 2–4 weeks) and post-weaning enrichment treatments were applied (described in^23^) to stimulate variation in cognitive development^24–26^. Briefly, enriched pens were larger (11.2 m^2^), with deep straw bedding and a range of enrichment materials that were frequently replenished. Barren pens were smaller (5.5 m^2^) and were minimally enriched, with wood shavings and mats for thermal comfort. Here, we focus on the correlations between cognitive test performance and later agonistic behaviour, rather than investigating the causes of cognitive differences. We define cognitive performance experimentally rather than directly comparing the early life treatments with aggressive outcomes, because i) we applied the treatments at the litter level and would require an unfeasibly large sample size to separate genetic, maternal and treatment effects on cognition, ii) enrichment may influence motivation as well as cognition^23^ and iii) socialisation and enrichment do not affect individuals equally^12,27^.

**Fig. 2:**
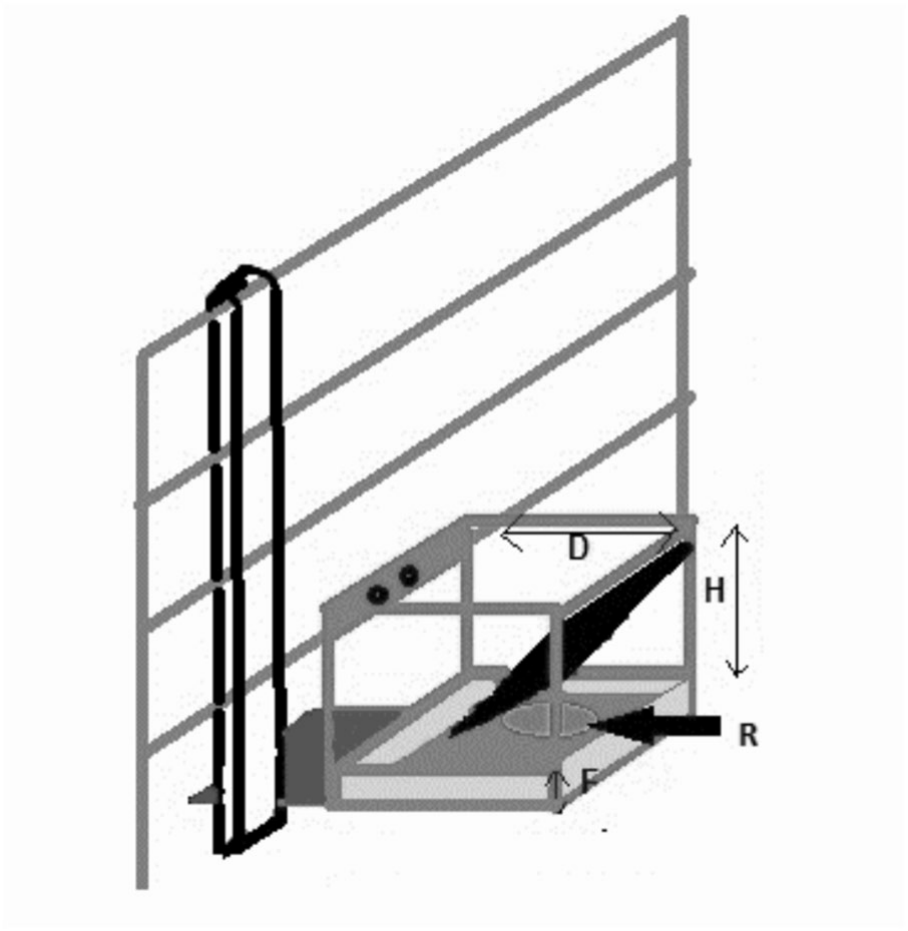
Image of goal box used for the spatial discrimination test. The internal dimensions of the box were 24 cm depth (D) and 24 cm height (H) and the box contained a 10 cm high false floor (F), underneath which an inaccessible reward was placed. R indicates the location of the accessible reward (above the false floor).

From weaning, pigs were habituated to humans in the pen. Gentling was applied gradually over 2 weeks, only moving to the next stage when pigs were willing to approach, starting with a person sitting in the pen, then standing and walking, then using a pig board, then touching the pigs’ head and body (to prepare for later lesion scoring). At 6 weeks of age, four test pigs per litter were moved to an experimental building and penned in litter groups. Each pen contained 4 familiar littermates (range 3–5) plus up to 5 additional littermates which were used as stimulus pigs in a social preference test (not analysed in this study). Stimulus pigs were removed from the group before training for the cognitive tests started at 8 weeks of age. Thereafter, group composition was stable until experimental regrouping at 14 weeks of age. From 6 to 9 weeks, pigs were gradually habituated to food rewards (a chocolate peanut and a slice of banana), handling and brief isolation from pen-mates. At 9 weeks, pigs were habituated to the test arena and goal box (Fig. 2).

### Spatial discrimination test

Each farrowing batch of litters was split into two cohorts and trained on alternate days. Left and right locations were assigned as positive (food reward) and negative (no food reward and a fan turned on) respectively and pigs were then allocated to positive as either left or right, balanced within sex and litter. Each pig had two pre-acquisition sessions of six trials, during which they learnt to open the goal box, and then five sessions of pseudorandomised training trials, containing a total of 36 trials. We avoided presenting more than two negative trials consecutively so pigs were not too discouraged. If pigs did not habituate or did not explore the box in the negative location with sufficient remaining trials to pass (12 trials), they were excluded. This was to confirm that avoiding the negative location was due to a learnt association rather than neophobia. We then measured learning speed from the trial during which the pig first opened the goal box in the negative location, i.e. had experienced both types of cue. Learning speed was measured in number of trials to reach the pass criterion.

In each trial, pigs were taken out of the arena by experimenters once they had: i) eaten the reward, ii) made a “no go” decision by looking towards the goal box and returning to the entry gate for three seconds, iii) exhibited signs of stress (loud or high-pitched vocalizations, attempting to escape from the arena), or iv) after 30 s. Between trials within a session, the pig left the arena for the time it took to replenish and/or move the goal box. The pass criterion was based on consistent correct decisions with a probability of p < 0.05, which equated to 10/12 consecutive correct decisions. A correct decision was “go” (opening the goal box) if the box was in the positive location, or “no go” (do not open the goal box) if the box was in the negative location.

Latency to open the goal box was recorded live using a stopwatch by an observer positioned at the entrance to the arena. In cases where it was unclear whether the pig had opened the goal box, the experimenter (positioned behind the goal box, as shown in figure S3) provided confirmation to the observer. Latency was recorded to the nearest second. While formal inter-observer reliability tests were not conducted between the four observers, all new observers were paired with an experienced scorer for initial sessions to ensure consistency in scoring. Additionally, observers were rotated across test groups and times of day to avoid systematic bias.

### Reversal learning

The reversal learning test took place during batches 3–8 (total 151 pigs) of which 102 demonstrated a consistent preference for the positive over the negative location in the spatial discrimination and then completed the reversal learning test. Data were collected live using a stopwatch, as was done in the spatial discrimination test.

Firstly, two goal boxes were presented concurrently, on the left and right of the arena. Pigs were given up to 3 refresher sessions of 6 trials each, for habituation to the second box, and to confirm that they remembered the reward location. The reversal learning phase followed, comprising 20 trials during which the positive and negative locations were reversed. In the first trial of the reversal learning test, once the pig had opened the goal box in the new negative location or after 30 s, the flap of the box in the new positive location was opened by the experimenter to reveal the reward. A pass result in the reversal learning test required pigs to approach the new positive location as a first choice, for 6/6 or 10/12 consecutive trials.

### Regrouping

At 14 weeks of age, the four pigs in each litter were ranked according to the outcome of the spatial discrimination and reversal learning tests. Points were allocated for: i) passing the spatial discrimination test (SDT), ii) above average learning speed in the SDT and iii) passing the reversal learning test, and then summed. The two highest scoring pigs were allocated to a “high test performance” group and the other two to a “low test performance” group. It was essential to balance groups based on litter as pigs perform more aggression towards unfamiliar pigs than littermates. Each pig was accompanied by a single littermate, when possible. Due to variations in litter sizes or pigs being removed from the trial due to health issues, four pigs moved with no littermate; 136 with 1 littermate; 31 with 2 littermates; and 4 moved with 3 littermates.

Pens were 6.1 × 2.35 m including a slatted dunging area, with a maximum of 16 pigs per pen, resulting in a space allowance of 1.02 m^2^/pig on average. Within each of 7 batches, males and females were equally distributed across pens and the body weight variation within each mixed pen was kept to a minimum. This resulted in 175 pigs being relocated across 14 groups (across 7 batches). Throughout the experiment, pigs were identified using spray paint (see figure S4). In the morning, prior to mixing, pigs were weighed to the nearest 0.5 kg and whilst in the weigh crate were given a new identification mark, to avoid having multiple pigs with the same ID in the same pen. Mean body weight and standard deviation were 62.4 ± 7.2 kg overall and within each pen, standard deviation varied from 3.78- 6.84 kg.

### Aggressive behaviour and skin lesions

Pens were video recorded for 5 h continuously immediately post-mixing. Mixing was carried out at midday after morning husbandry in the building where the pigs were housed, to avoid further disruption., and behavioural data were obtained using “The Observer XT15 and XT16” software (Noldus, Wageningen, NL). This period has been identified as an appropriate window to capture the peak of aggression after regrouping^28^. For all aggressive interactions, the identity of the giver and receiver and the type of behaviour was recorded by a single observer (Table 2) who was blind to the cognitive performance of each pig and the category of the pen (high/low cognitive performance). The data for all 14 pens were scored in 30-min segments, rather than scoring the batches sequentially, to ensure any variation in scoring would not be systematic across batches. The use of a single highly trained observer was intended to avoid inter-observer variation. Nevertheless, in prior studies of post-mixing aggression in pigs, high levels of agreement between observers are reported, indicating a high level of consistency in use of this ethogram^29,30^. In the current study, the observer was trained by experienced colleagues (the same observers in the studies cited here). The same observer also counted all fresh skin lesions in the anterior, central, and posterior body regions immediately before group mixing, 24 h post-mixing, and at one week and two weeks post mixing. Lesions were considered recent if they were vivid red or recently scabbed^11^. All lesions observed were counted equally, regardless of size and severity. For lesion counting, it was not possible to perform intra-observer reliability testing across batches because this was carried out live. To avoid variation in how lesions were counted, the observer was trained by colleagues experienced in lesion scoring and followed a protocol used on the pig unit for previous studies. Skin lesion counts were calculated by subtracting lesions in the morning before mixing from lesions 24 h later. Negative numbers were converted to zero.

**Table 2:** Ethogram of agonistic behaviour post-regrouping.

	Behaviour		Point or State
Display	Heads up	Both pigs lift their snouts, so they are pointed in the air and alongside each other	S
	Parallel walking	Both pigs walk simultaneously with the shoulders next to each other, terminated when pigs change position or stop walking	S
Non-damaging aggression	Head-knock	A rapid thrust upward or side to side with the head or snout against any part of another pig. Contacts the other pig with force	P
	Lunge	Sudden head movement towards another pig, focal pig has mouth open or closed, physical contact may or not be made but does not bite recipient pig	P
	Shove	A rapid and sudden movement of the body towards another pig, which results in the other pig moving position	P
	Flick	Sudden sideways head movement directed towards another pig but not forceful	P
	*“Shove and flick” were very subtle, brief and time-consuming to identify and were not scored after 2 h post-regrouping*		
	Pushing	Head or shoulder is used to move the other aside by applying pressure, for a minimum of 1 s, terminated when physical contact is lost	S
	Single bite	Pig bites the head, neck, or body of another. Bite must be observed to land, or the recipient seen/heard to respond as if bitten, otherwise counted as a lunge Once a fighting bout starts, individual bites are not recorded	P
Unilateral aggression	Bullying (biting or chasing)	A rapid sequence of bites is delivered without the opponent retaliating, or pig pursues opponent after they have retreated and delivers further bites	S
Mutual fighting	Fighting	A bite or series of bites is immediately (within 3 s) reciprocated with more than one bite. Fight continues if at least one bite per 3 s is delivered. Initiator is the pig which delivers the first bite or bite which is reciprocated Fight ends when aggressive acts cease for 60 s or more	S
	Rest during fight	Fighting stops and is recorded as “rest” if few (< 1 per 3 s) or no bites were attempted. Pigs remain in contact but not pushing	S
	Retreat	A submissive behaviour of an involved pig, i.e., turning away with a sharp head-tilt movement, voluntary displacement from a location (except from feeder) or fleeing > 1 m and not delivering aggression within 3 s	P
Other behaviour	Mounting	A standing pig lifts its two front legs and puts the two legs or its breast on any part of the body or head of another pig	P
	Other	Default state including eating, lying, sitting, standing, walking, rooting or gentle contact with pen mates, including nosing and climbing	S

### Social network analysis

In line with previous studies of aggression networks in indoor pigs^14,21^, we constructed four networks to reflect the range of agonistic behaviour (see Table 2):

1. ALL networks contained all agonistic behaviour (damaging and non-damaging).
2. UNI networks contained unidirectional, non-reciprocated, damaging aggression.
3. FIGHT networks contained mutual fighting.
4. WIN networks contained fights with decided outcomes, i.e. one pig clearly signalled defeat. Edges were directed from winner to loser.

ALL, UNI, and FIGHT networks were weighted by duration, with point behaviours assigned a weight of one second and then summed with the duration of state behaviours. WIN networks were weighted by frequency of fights. Networks were directed, except for FIGHT networks.

The following centrality measures were analysed: degree centrality (in, out and all degree), betweenness centrality and eigenvector centrality. Eigenvector centrality is an undirected measure, so we analyse this in the context of FIGHT networks only.

Although the study was designed to have equal group size and familiarity (i.e. balanced litter distribution across groups), it was not possible to maintain consistent network size and familiarity in this experiment due to health and fertility reasons. Therefore, we applied corrections to the calculations of degree, betweenness and eigenvector centrality to account for group size (see supplementary methods). Graphs were created using the package “igraph”^31^ and collapsed using “simplify” from the package “sna”^32^to remove multiple edges between the same dyad. Centrality was extracted using the functions “degree”, “betweenness” and “eigen_centrality” in igraph.

### Statistical analysis

Analysis was carried out in R version 4.3.1^33^.

The outcomes of the cognitive tests were i) number of trials required to score 10/12 consecutive correct choices in the spatial discrimination task and ii) pass or fail in the reversal learning test. Passing the reversal learning test required 6/6 or 10/12 consecutive correct choices. A score of 36 was assigned to pigs which completed but did not pass the spatial discrimination test. The number of trials to pass the spatial discrimination task was therefore bounded at 12–36. The distribution was moderately skewed by the 33% of pigs which passed in the first 12 trials, so learning speed was analysed as a categorical trait (“slow” = fail or pass in > 17 trials, “fast” = pass in ≤ 17 trials, where 17 trials was the median number of trials required to pass).

### Analysis of social networks and skin lesions

#### Null model

Mixed models using lme4^34^ (LMM for continuous network traits, GLMM with a poisson or binomial link for count or binary outcomes) were used to account for fixed and random effects. Assumptions of linear models were assessed by visual inspection of residuals using the r package “performance”^35^ and assessment of variance inflation factor to detect multicollinearity. Where residuals of the final null model violated the assumptions of the model, outcomes were transformed or categorised (see supplementary results, table S3).

A null (partial) model was constructed, without cognitive performance measures. Centrality measures and skin lesion counts were used as response variables in separate models with the fixed effects of sex, body weight and network size, defined according to the number of non-littermates in the pen (see supplementary methods), which varied from 6 to 14, mean 10.8, s.d. = 2.09. Random effects were network ID (14 levels), and litter (46 levels).

Terms identified as candidates for dropping from the model were checked by performing likelihood ratio tests (LRT; using the chi square distribution) or Wald (F) tests of the effect size (scaled using estimated standard error), using the “anova” function, to assess whether the complex model was better at explaining variability in the data than the model without the term. The statistical significance of random effects was evaluated using LRT tests, and by inspecting the change in model fit statistics (AIC and RMSE) associated with inclusion of random effects.

#### Full model

Each cognitive outcome was included as a fixed effect in a full model and their significance was assessed using Wald (F) tests and based on comparing the model fit statistics with those of the null model. Of the 175 pigs in the regrouping treatment, 50 had not completed the spatial discrimination and 73 had not completed the reversal learning test. Inclusion in each cognitive test was evaluated as a binary fixed effect, before evaluating the cognitive outcomes. Cognitive outcomes were a) learning speed in the discrimination task (fast/slow) and b) pass/fail in the reversal learning test.

## Results

### Spatial discrimination and reversal learning

**Fig. 3:**
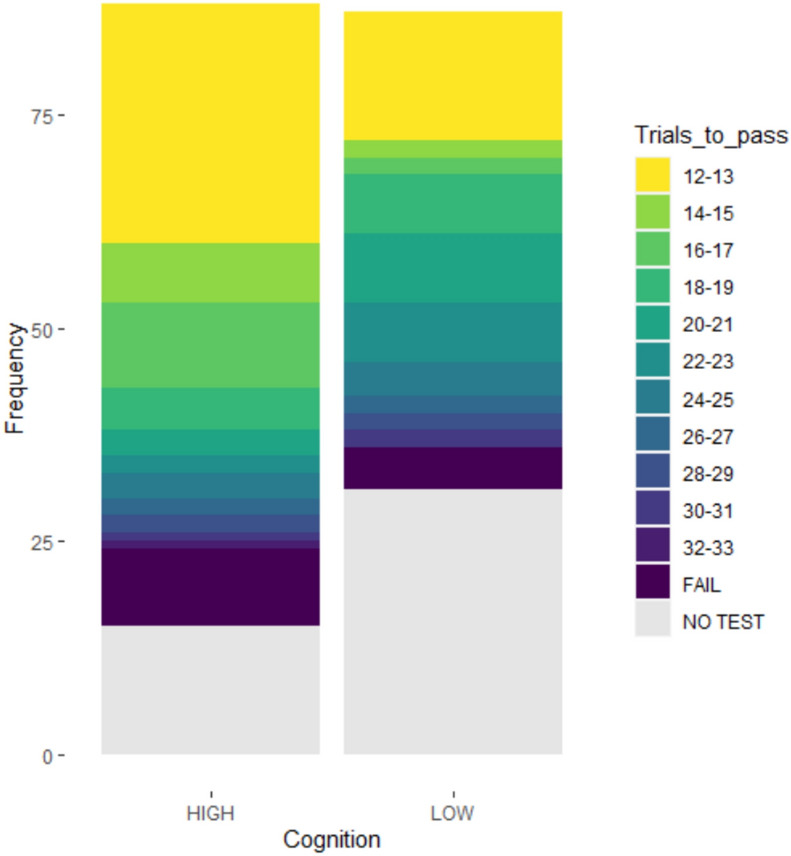
Results of the spatial discrimination task, split by “high” (higher scoring in cognitive tests compared to littermates) and “low” (lower performance in cognitive tests compared to littermates) regrouping pens. Pigs which did not complete the test are indicated in grey, with learning speed varying from yellow (fastest) to purple (slowest), using the r colour scale “viridis.”.

Pigs took 12 to 33 trials to achieve the pass criterion (10/12 correct choices). The distribution of the number of trials to pass the task was negatively skewed, with 42/141 (30%) pigs passing in the minimum possible number of trials (Fig. 3). Ten pigs completed all trials but failed to reach the pass criterion.

Inclusion in the spatial discrimination test did not significantly affect centrality outcomes, whereas inclusion in the reversal learning test had a slightly negative effect on eigenvector centrality (LS means and confidence intervals were: reversal: 0.20, 0.18–0.21, no reversal: 0.25, 0.23–0.27; F = 5.22, p = 0.021). For full results see tables S5 and S6. Differences are reported as significant if p < 0.05 and a tendency if p < 0.1.

A total of 51 pigs (out of 102) passed the reversal learning test by meeting the criteria: i) 10/12 consecutive correct trials (33/102, 32%); or ii) 6/6 consecutive correct trials (47/102, 46%) within 20 trials. These outcome measures were positively correlated (r = 0.580, p < 0.001) and 29 pigs passed according to both criteria. Most pigs which reached the pass criteria required 19–20 trials to pass (64% of those which reached 6/6 correct, 70% of those which reached 10/12 correct). Reversal learning speed was not analysed as a predictor of centrality in aggression networks or skin lesions because of a lack of variation.

### Agonistic behaviour

In the 5 h after regrouping, pigs each spent a median of 633 s (35—2982 s) performing agonistic behaviour and 602 s (86–2963 s) receiving agonistic behaviour. For summary statistics of individual agonistic behaviours, see supplementary results, tables S1-S2.

### Social network characteristics

We measured unweighted and weighted networks (for descriptive statistics of all network outcomes, see table S3), however there was little variation in unweighted degree centrality in networks containing all agonistic interactions (ALL). In almost all pens each pig exchanged some form of agonistic behaviour with all other non-littermates, 81% (142/175), of the pigs had an unweighted degree centrality of 1 (range 0.785–1), so we could not statistically analyse the causes of variation in unweighted degree centrality.

Weighted measures were considered redundant if they correlated at > 0.8 with unweighted centrality traits in the same network (table S4). These were: In WIN networks, indegree and outdegree centrality, and in UNI networks, betweenness centrality. For these networks, only unweighted measures are reported.

### The effects of non-cognitive factors on individual centrality

**Fig. 4:**
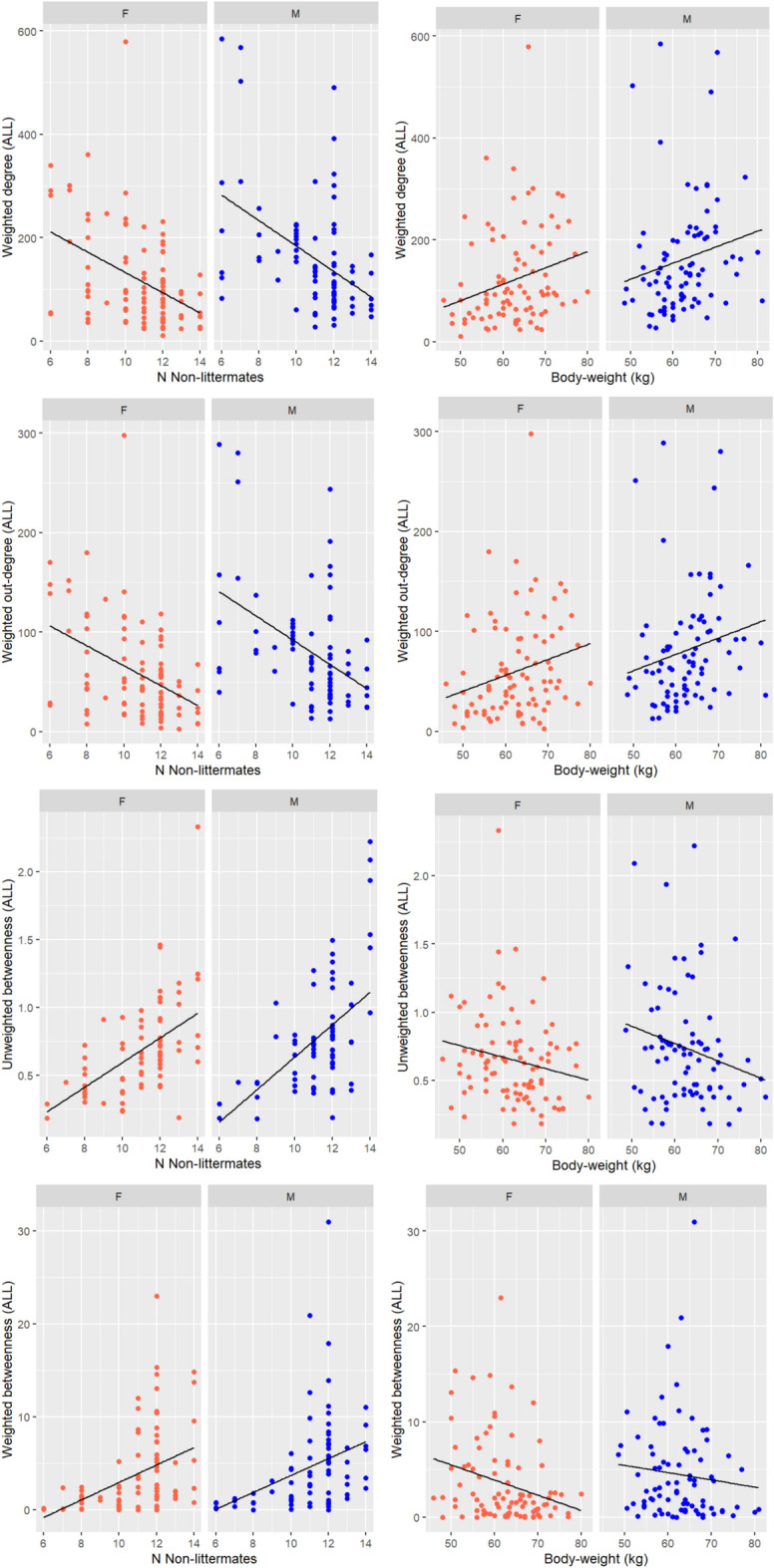
Scatterplots showing the relationship between centrality in ALL networks on the y axis (weighted degree, weighted outdegree, betweenness, weighted betweenness) and on the x-axis: number of non-littermates (left) or body weight (right). Each plot is split into females (red dots) and males (blue dots).

The effects of sex, body weight and network size on individual centrality are summarised in Fig. 4.

#### Sex

In ALL networks, weighted degree and weighted outdegree centrality were higher in males than females (Table 3). In UNI networks, males had a higher unweighted outdegree centrality than females. Weighted degree was greater for females, which was driven by indegree more than outdegree, i.e. females received more unilateral aggression than males (number of opponents and duration). In FIGHT networks, unweighted degree was unaffected by sex, but males had greater weighted degree centrality than females. Unweighted betweenness centrality was not affected by sex but weighted betweenness was higher in males. In WIN networks, males had a non-significant tendency towards greater outdegree centrality than females (F = 3.36, p = 0.069) and had lower eigenvector centrality compared with females (Table 4). Betweenness centrality was not affected by sex.

**Table 3:** Effects of non-cognitive traits on individual centrality in aggression networks.

Network	Centrality	Male mean, CI	Female mean, CI	F/Χ^2^	Weight effect size, CI	F/Χ^2^	Network size effect size, CI	F/Χ^2^
ALL	Weighted degree	135 (126–144)	90 (84–96)	18.1**	0.02 (0.02- 0.03)	12.9**	−0.12 (−0.13- −0.10)	32.5**
	Weighted indegree	55 (51–59)	56 (52–60)	0.03	0.01 (0.00- 0.02)	1.11	0.04 (0.02- 0.07)	2.84
	Weighted outdegree	67 (62- 72)	42 (39- 45)	18.2**	1.02 (1.02- 1.03)	10.0**	0.87 (0.84- 0.89)	29.1**
	Betweenness	0.682 (0.653- 0.712)	0.63 (0.606- 0.659)	1.54	−0.003 (−0.005- −0.002)	4.37*	0.059 (0.053- 0.065)	97.6**
	Weighted betweenness	2.78 (2.49- 5.09)	2.16 (1.93- 4.40)	2.74	−0.019 (−0.026- −0.011)	6.17*	0.223 (0.191- 0.255)	58.8**
UNI	Degree^†^	OR: 0.92 (0.53- 1.22)		−0.06	1.00 (0.97- 1.03)	−0.01	OR: 0.79 (0.71- 0.87)	−6.10*
	Indegree	0.78 (75–82)	0.74 (70–77)	3.35	−0.000 (−0.001- 0.002)	0.01	0.01 (0.00- 0.02)	2.46
	Outdegree^†^	OR:3.7 (2.46- 5.48)		−3.97*	1.00 (0.97- 1.02)	−0.01	OR: 0.80 (0.72- 0.88)	−6.16*
	Weighted degree	9.6 (9.0- 10.20)	12.1 (11.4- 12.8)	6.67*	0.007 (0.001- 0.012)	1.41*	−0.010 (−0.030- 0.009)	0.28
	Weighted indegree	5.0 (4.6- 5.4)	5.4 (5.0–5.8)	0.68	1.00 (0.99- 1.01)	0	0.96 (0.92- 1.00)	1.19
	Weighted outdegree	4.6 (4.1–5.1)	4.2 (3.8–4.7)	0.32	0.01 (0.00- 0.01)	1.41	−0.01 (−0.03- 0.01)	0.28
	Betweenness	1.22 (1.12- 1.31)	1.11 (1.03- 1.20)	0.70	0.00 (−0.01- 0.00)	0.94	0.09 (0.07- 0.10)	34.6**
FIGHT	Degree	0.65 (0.63- 0.67)	0.65 0.63- 0.67	0.001	−0.002 (−0.003- 0.002)	−0.002	−0.013 (−0.020- −0.006)	3.30
	Weighted degree	73 (68- 78)	52 (48- 56)	−76.8**	0.004 (0.001- 0.009)	5.35*	0.150 (0.082- 0.238)	15.0**
	Betweenness^†^	OR: 0.58 (0.40- 0.84)		−2.25	1.03 (1.01- 1.06)	−0.09	1.25 (1.13- 1.38)	−2.94
	Weighted betweenness^†^	OR 2.54 (1.75- 3.68)		−6.53*	0.99 (0.97- 1.02)	−1.64	1.17 (1.06- 1.29)	−5.53*
WIN	Indegree	0.37 (0.35- 0.39)	0.35 (0.33- 0.37)	0.33	0.004 (0.002- 0.006)	4.23*	0.007 (−0.003- 0.010)	0.27
	Outdegree	0.38 (0.35–0.40)	0.31 (0.29- 0.34)	3.36	0.001 (−0.001 – 0.003)	0.247	−0.005 (−0.011- 0.001)	0.69
	Eigenvector	0.16 (0.14- 0.17)	0.28 (0.27- 0.30)	33.4**	−0.001 (−0.002- 0.000)	0.40	−0.002 (−0.007 – 0.002)	0.34

CI = confidence intervals, F/Χ^2^ refers to the test statistic of Wald or LRT tests respectively. Results are back-transformed least-squared means and confidence intervals derived from lmm, which were estimated for data transformed using log(x + 1) using the calculation e^(mean ± s.e.) −1. † Results were derived from GLMs, log-it values are transformed to odds ratios (OR) and confidence intervals are estimated using e^(OR ± s.e.).

**Table 4:** Effects of cognitive traits on individual centrality in aggression networks.

	Centrality	SDT_fast	SDT_slow	Χ^2^ p	Pass reversal	Fail reversal	Χ^2^ p
ALL	Weighted degree	118 (108–129)	109 (100- 119)	0.53, 0.467	106 (96- 117)	108 (98–119)	0.06, 0.812
	Weighted indegree	53 (46–61)	57 (50- 65)	0.36, 0.551	47 (42–53)	51 (46- 58)	0.40, 0.526
	Weighted outdegree	57 (52- 63)	51 (46- 57)	0.64, 0.424	51 (45- 57)	51 (45- 57)	0.00, 0.970
	Betweenness	1.25(1.22- 1.29)	1.24 (1.20- 1.28)	0.12, 0.726	54.1 (47.7- 61.3)	54.6 (48.2- 61.9)	0, 1
	Weighted betweenness	2.49 (2.18- 2.83)	2.35 (2.05- 2.69)	0.12, 0.730	2.78 (2.40- 3.20)	2.36 (2.11- 2.84)	0.40, 0.529
UNI	Degree^†^	OR: 0.80 (0.53- 1.22)		−0.27, 0.597	OR: 0.78 (0.50- 1.21)		−0.32, 0.573
	Indegree	0.79 (0.77- 0.81)	0.74 (0.72- 0.77)	2.38, 0.123	0.79 (0.76- 0.81)	0.75 (0.72- 0.77)	1.59, 0.208
	Outdegree^†^	OR: 1.11 (0.72- 1.73)		−0.06, 0.810	OR: 1.99 (1.22- 3.23)		−2.04, 0.153
	Weighted degree	12 (10- 13)	10 (9–11)	2.71, 0.100	11 (10–13)	9 (8–10)	5.33, 0.021*
	Weighted indegree	5.31 (4.84- 5.83)	4.66 (4.24- 5.14)	1.11, 0.29	5.70 (5.14- 6.31)	4.81 (4.34- 5.32)	1.59, 0.21
	Weighted outdegree	3.9 (3.4- 4.5)	4.1 (3.5- 4.8)	0.080, 0.778	5.0 (4.2- 5.8)	3.6 (3.1- 4.3)	2.45, 0.117
	Betweenness	1.17 (1.07- 1.28)	0.76 (1.03- 1.25)	0.05, 0.824	1.27 (1.14- 1.41)	1.22 (1.07- 1.28)	0.07, 0.798
FIGHT	Degree	0.64 (0.61- 0.67)	0.63 0.60- 0.66	0.04, 0.841	0.64 (0.60- 0.67)	0.66 (0.62- 0.69)	0.21, 0.65
	Weighted degree	68 (61- 74)	62 (56- 69)	0.42, 0.519	65 (58- 73)	60 (52–67)	0.53, 0.466
	Betweenness^†^	OR: 0.29 (0.28–0.30)		−0.02, 0.885	OR: 0.90 (0.57- 1.43)		0.05, 0.818
	Weighted betweenness^†^	OR: 1.23 (0.83- 1.96)		−0.33, 0.566	OR: 1.48 (0.93- 2.36)		−0.72, 0.397
WIN	Indegree	0.36 0.34- 0.38	0.36 (0.34- 0.38)	0.00, 0.985	0.39 (0.37- 0.42)	0.33 (0.30- 0.35)	3.41, 0.068
	Outdegree	0.35 (0.32- 0.38)	0.34 (0.31- 0.37)	0.06, 0.815	0.39 (0.36- 0.43)	0.31 (0.28- 0.34)	3.66, 0.056
	Eigenvector	0.20 (0.18- 0.22)	0.22 (0.20- 0.24)	0.32, 0.574	0.19 (0.17- 0.21)	0.21 (0.19- 0.23)	0.53, 0.470

SDT- Spatial discrimination task. Results are back-transformed least-squared means and confidence intervals derived from lmm, which were estimated for data transformed using log(x + 1) using the calculation e^(mean ± s.e.) −1. ^†^ Results were derived from GLMs, log-it values are transformed to odds ratios (OR) and confidence intervals are estimated using e^(OR ± s.e.).

#### Body weight

In ALL networks, as body weight increased, weighted degree centrality and outdegree increased, while unweighted and weighted betweenness decreased (Table 3). In UNI networks and in FIGHT networks, increasing body weight increased weighted degree centrality only. Unweighted indegree centrality in WIN networks was positively associated with body weight, indicating that heavier pigs tended to lose fights against more opponents. For every 20 kg rise in body weight, pigs had an increase of 0.08 in indegree centrality (equivalent to losing against one more opponent in an average pen with 12 non-littermates). This relationship was not significant in weighted WIN networks, (F = 3.10, p = 0.08). Outdegree and eigenvector centrality were not affected by body weight.

#### Network size

Network size affected centrality despite ALL, UNI and FIGHT networks already having been adjusted to account for network size. In ALL networks, increasing network size decreased weighted degree centrality but increased weighted outdegree centrality, and unweighted and weighted betweenness centrality. In UNI networks, increasing network size decreased unweighted degree centrality and outdegree centrality and increased betweenness centrality. In FIGHT networks, increasing network size increased weighted degree and weighted betweenness centrality. Network size did not affect centrality in WIN networks.

### Associations between cognitive test performance and regrouping aggression

**Fig. 5:**
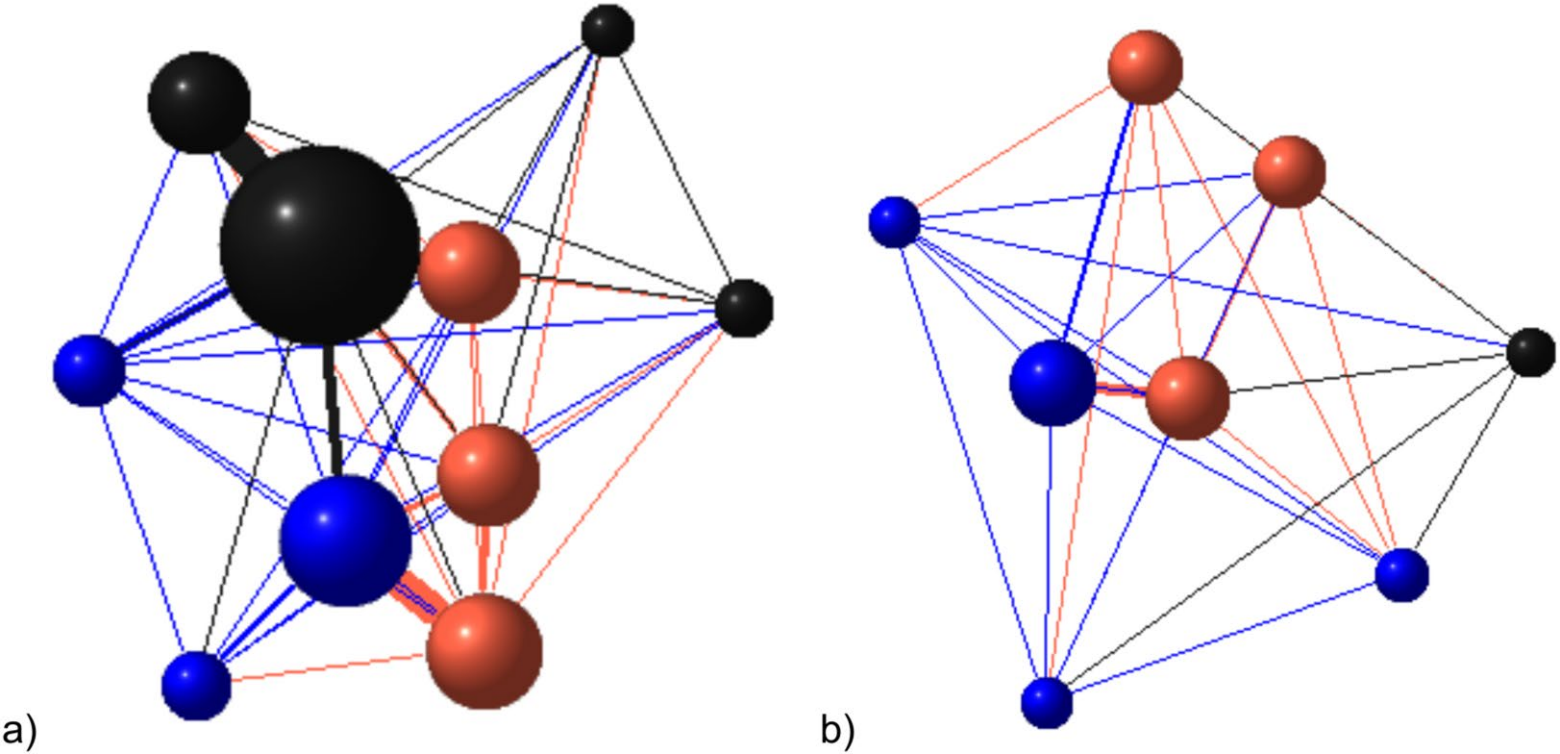
Model to show relationship between reversal learning outcome and weighted degree in UNILATERAL networks generated using data from a) batch 3 pen 1 and b) batch 4 pen 2. Node size is proportional to weighted degree centrality, edge width is proportional to edge weight (total duration of unilateral aggression between each dyad). The colour of the node reflects performance in the reversal learning test (red = pass, blue = fail, black = no test). Weighted degree centrality is slightly higher in pigs which passed the reversal learning test, so the red nodes are slightly larger than the blue nodes.

In ALL and FIGHT networks, the results of the cognitive tests were not associated with centrality. In UNI networks, learning speed had no effect on individual centrality, whereas pigs which passed the reversal test engaged in more unilateral aggression (weighted degree) than pigs which failed the test (Table 4; Fig. 5).

In WIN networks, there was a tendency for pigs which passed the reversal learning test to have both a higher indegree centrality and a higher outdegree centrality than those which failed. Eigenvector centrality was not associated with cognitive performance.

Performance in cognitive tests had a small effect on the number of skin lesions received at 24 h, 1 week or 2 weeks after mixing (Table 5).

**Table 5:** Effect of performance in cognitive tests on lesion counts after group mixing.

Learning speed (categorical)	FAST (n = 64 pigs)	SLOW (n = 61 pigs)	Χ^2^	P
SL 24 h				
Anterior	75 (62–91)	79 (65–95)	3.94	0.047
Central	7 (5–11)	8 (5–12)	1.77	0.18
Posterior	4 (3–7)	3 (2–4)	19.9	< 0.001
SL 1 week				
Anterior	12 (10–15)	9 (8–12)	16.1	< 0.001
Central (YES/NO)	OR 0.75 (0.50–1.12)		0.52	0.47
Posterior (YES/NO)	OR 1.82 (1.26–2.62)		2.71	0.10
SL 2 weeks				
Anterior	9 (7–12)	9 (7–11)	0.07	0.79
Central (YES/NO)	OR 1.13 (0.74- 1.74)		0.09	0.77
Posterior (YES/NO)	OR 0.45 (0.28- 0.72)		3.27	0.07

Pass reversal learning test	PASS	FAIL		
SL 24 h				
Anterior	81 (62–106)	75 (57–98)	8.49	0.004
Central	8 (5–10)	7 (6–12)	3.41	0.06
Posterior	3 (2–4)	3 (2–5)	1.14	0.29
SL 1 week				
Anterior	12 (9–16)	10 (8–14)	5.21	0.02
Central (YES/NO)	OR 0.50 (0.32–0.78)		2.44	0.12
Posterior (YES/NO)	OR 1.70 (1.11–2.62)		1.59	0.21
SL 2 weeks				
Anterior	9 (7–12)	9 (7–12)	0.04	0.84
Central (YES/NO)	OR 0.68 (0.44- 1.06)		0.76	0.38
Posterior (YES/NO)	OR 1.35 (0.85–2.13)		0.43	0.51

Skin lesions (SL) were counted at 24 h, 1 week and 2 weeks following mixing. Lesions counted before group mixing were deducted from lesions counted at 24 h, while counts at 7 days and 14 days only included fresh lesions assumed to have occurred within 24 h. Values are least squared means and 95% confidence intervals derived from glmm, back transformed using “lsmeans”. Values are rounded to the nearest whole number of lesions.

Fast learners in the social discrimination test received fewer anterior lesions and more posterior lesions in the acute 24 h after mixing than slow learners. They also received more anterior lesions at 1 week after mixing. Pigs that passed the reversal learning test had more anterior lesions at 24 h and 1 week after mixing than those that failed the test. By 2 weeks post-mixing there were no significant associations between cognitive performance and skin lesions.

## Discussion

This study provides evidence that pigs which vary in cognitive performance have differential outcomes in their agonistic behaviour post mixing.

We predicted that pigs with higher cognitive performance would receive less agonistic behaviour and avoid prolonged or repeated aggressive interactions. We also predicted that cognitive ability would decrease the cost of aggression in terms of skin lesions, which may be apparent during the acute, dominance-related fighting, or later at 1 or 2 weeks, reflecting undecided or unstable dominance relationships and conflict over resources. However, we found no relationship between cognitive performance and the total weighted all-degree, in-degree or out-degree in all agonistic behaviour networks (ALL). Agonistic behaviour that does not involve physical injury, such as parallel walking and pushing may be necessary for pigs to learn about unfamiliar opponents, and because they are low cost, learning to avoid these sorts of interactions is not ecologically relevant. Furthermore, in contrast to our predictions, passing the reversal learning test was associated with higher weighted degree centrality in unilateral aggression networks (unreciprocated aggression) and was related to receiving more skin lesions. There was no evidence that these pigs specifically acted as the givers or receivers of unilateral aggression.

In line with predictions that pigs with better cognitive performance would receive fewer skin lesions, faster learning in the spatial discrimination task was associated with pigs receiving fewer skin lesions. However, this difference was reversed after one week and better performance in the reversal learning test was associated with receiving more skin lesions in the anterior body region. Similarly, other social network studies of pigs have found that lower centrality in aggression networks post-mixing did not translate to receiving fewer skin lesions 3 weeks post mixing^11,14^. It is possible that pigs which learnt the spatial association faster were more food-motivated than pigs which learnt the task slowly. In the acute period of fighting after mixing, agonistic behaviour occurs immediately when pigs enter the pen and is unrelated to feeder access. However, aggression that occurs later once dominance relationships have largely been settled are often related to feeder use, so these pigs could have been more involved in food competition at 1 week post mixing.

We predicted that pigs with better cognitive performance would have better fighting success and higher eigenvector centrality. Our results partially support this; pigs with better performance in the reversal learning test had a tendency towards higher indegree and outdegree centrality in fighting networks directed by outcome, suggesting that they both won and lost against more opponents. The correlation between successful reversal learning performance, being involved in more unilateral aggression and being involved in fights with a decisive ending, both as winners and losers, suggests a possible link between cognitive performance and skilful fighting, including both offensive and defensive behaviour^5^.

Novel tests of social cognition may be required to access the cognitive domains relevant to aggression. Measuring assessment ability using repeated contests poses clear welfare problems^36^ and assays of cognition which involve pain and stress can themselves inhibit learning^37^, therefore reward based learning is preferred. However, prior studies had limited success conditioning pigs to prefer one unfamiliar conspecific over another using food rewards^38^, which we also failed to achieve in a suitable time frame in pilot tests for this study.

The association between passing the reversal learning test and receiving more anterior skin lesions could indicate that rather than selecting opponents based on the likelihood of winning, they select opponents of similar or slightly higher fighting ability based on the potential gain in dominance. To understand whether cognitive ability enhanced pigs’ success in winning contests, as we found in dyadic contests^23^ and resulted in pigs’ occupying higher dominance ranks, dominance rating systems could be applied, such as Glicko-2, which takes into account the focal pig’s fighting success, the fighting success of their opponents, and the confidence in this estimate^39^.

The effect of network size on aggression was interesting and persisted even though we attempted to normalise social network outcomes by dividing by the total number of non-littermates. With more potential opponents, pigs engaged in more mutual fighting and less unilateral aggression. This may reflect pigs’ greater capacity to remember previous interactions with individuals in smaller groups, since brief unilateral aggression is used to maintain dominance relationships without escalating to fighting. Keeping pigs in large, homogenous groups is in contrast with the natural history of pigs, which live in matrilineal societies in which adult males are largely solitary^40^. Weighted betweenness centrality was increased by network size in every type of network. In larger groups, indirect connections become more important and betweenness centrality may be more sensitive to cognitive differences if pigs were evaluated in large groups.

In unweighted, directed networks, males were more central (degree) than females, i.e. they directed aggression towards a greater number of others. Males also performed more aggression overall, as is characteristic of pig aggression^41^. Total aggression received (weighted indegree) was not affected by sex. The influence of sex on aggression may have limited our statistical power to detect more subtle influences of cognition, so testing cognitive effects on aggression in single sex groups may be more elucidating. Social network analysis of pigs has focussed on single sex groups of females or castrated males^19,21,42^. With pressure to eliminate castration in the EU, management changes must consider solutions to reduce aggressive behaviour between intact males. In this study the males were not castrated. Whilst reflecting the natural state of the animals, this likely resulted in larger sex differences compared to females than would have occurred if the males had been castrated. We did not have the capacity to test cognitive ability in large enough numbers to form balanced, single sex groups. Navigating mixed-sex groups could also be less cognitively demanding and therefore less sensitive to individual variation than single sex groups, because pigs can easily evaluate the sex of their opponent, compared with other aspects of fighting ability, such as size.

Female pigs had higher eigenvector centrality than males in WIN networks, demonstrating that females were more likely to engage in conclusive fights against pigs which themselves had fights with conclusive outcomes. Persisting in a contest to a clear outcome may be more advantageous for females because the cost of fighting is lower in contests between females than males. Surprisingly, sex did not affect indegree or outdegree in WIN networks, which may reflect a tendency for mutual fighting to occur primarily between individuals of the same sex.

Pigs with a higher body weight performed more aggression as shown in prior studies of regrouping aggression^41,43^. Indegree (losing fights) was positively associated with body weight in WIN networks, which is unexpected since smaller pigs are more likely to lose dyadic contests^44^. Higher body weight was associated with lower betweenness centrality in ALL networks, indicating that heavier pigs were less likely to connect individuals which did not directly interact. Larger pigs are more likely to be part of a fighting clique^14^ and may have low betweenness because they fight with others that are already well connected in the network. Dynamic network analysis of post-grouping aggression would lead to a better understanding of the direction of causality between betweenness, body weight, feeding behaviour and dominance.

A limitation of our experimental design was the necessity to train pigs between weaning and testing aggression at a commercially relevant age and body size, which led to an erosion from the initial sample size, similar to other studies involving spatial discrimination and reversal learning^12^. Pigs were regrouped even if they had not completed the cognitive tasks, to balance pen size and familiarity as far as possible. Therefore, social networks contained pigs which had no data for reversal learning.

We also note that the limitations on pigs’ ability to avoid aggression in a confined environment may mask individual strengths in learning and memory. Studies suggest that reversal learning performance is enhanced in species with greater social complexity and may allow wild animals to live in larger groups^45^ but intensely housed pigs have little choice over their own behaviour. This is an important finding, since in a confined pen of pigs kept at commercial stocking density, even pigs with higher cognitive ability may not have the space or opportunity to escape from attacks (figure S4). Testing pigs in larger or more complex environments may allow us to evaluate if pigs differ in their ability to use spatial skills to navigate both their spatial and social environment^46^.

We hypothesised that individual differences in cognitive ability would account for some of the unexplained variation in how pigs apportion aggression after group mixing. Most pigs successfully completed the spatial discrimination task, but their learning speed in this task did not predict centrality in social networks. Reversal learning performance had a minor effect on centrality in unweighted networks. We cannot over-interpret the importance of this result due to the large number of outcome measures evaluated and the limitation that only 51 pigs completed the reversal learning task. For future work, adapting the reversal learning test to extend the testing period may yield data on a greater proportion of pigs.

It remains to be seen how cognitive ability affects pigs’ sequential decisions, and the next step in the understanding of centrality measures such as betweenness and eigenvector centrality could include dynamic network analysis. The development of social cognitive tests that could be applied in a short time frame before mixing could elucidate cognitive domains we were not able to test in this study, such as communication, social discrimination, and individual recognition. We adapted McLeman et al*.*^38^ to measure pigs’ ability to discriminate unfamiliar conspecifics by size, however the time required was too long to habituate and train sufficient numbers of pigs to form replicated, balanced groups and then test aggression at a commercially-relevant stage (growing pigs).

In conclusion, the results of this study show that the performance of pigs in non-social cognitive tests does influence their role in group level aggression post mixing. However, the cognitive skills measured in these tests did not function as we had expected in protecting them from the costs of aggression. Since pigs can make associations between location and rewards and learn quickly to update information when cues are reversed, management strategies to reduce aggression could harness this capacity. This could include the use of more complex environments with multiple feeder locations, to reduce competition and allow defeated animals to avoid aggressors.

## Supplementary Information

Supplementary information 1.

Supplementary information 2.

Supplementary information 3.

Supplementary information 4.

Supplementary information 5.

## Data Availability

Data and R code associated with this paper are available on request from Lucy Oldham (lucy.oldham@sruc.ac.uk).
