# Supplementary material for: Cognitive and non-cognitive factors predict pigs’ positions in an aggression social network
Source: Sci Rep. 2025 May 20;15:17439. doi: 10.1038/s41598-025-02023-w (PMC12092641; doi:10.1038/s41598-025-02023-w)
Supplement: Supplementary file 1 — Supplementary information 1. [file 41598_2025_2023_MOESM1_ESM.pdf]

Cognitive and non-cognitive factors predict pigs' positions in an aggression social network

Supplementary materials

Supplementary methods

Network adjustments to account for network size and familiarity

Degree was adjusted according to pen size and familiarity by dividing by the number of non-littermates in the pen. For WIN networks, fights between littermates were included, because the number of proportion of littermates in the pen did not significantly influence the likelihood of winning fights . Betweenness was adjusted for pen size and familiarity by first calculating a theoretical maximum, i.e. the number of non-sibling dyads which did not include the focal pig.

Duration of sibling-directed aggression was concentrated such that most pigs performed little to no sibling-directed aggression (mean= 20.5s, median= 12.5 seconds), whilst 15 pigs performed one minute or longer of sibling-directed aggression. Sibling-directed aggression accounted for, on average a median of 2.4% (IQR 0.6-5.1%) of the total aggression and was weakly correlated with number of siblings as a proportion of the conspecifics in the pen (r_s= 0.20, p=0.009). The total duration of sibling-directed aggression was not significantly correlated with aggression towards non-siblings (r_s=0.011 p = 0.88). Aggression between siblings was much less likely to be injurious than aggression between non-siblings; the median proportion of injurious aggression between siblings was 21% (IQR 0-62%), compared to 64% (IQR 52-75%) between non-siblings. Due to the influence of familiarity on the frequency and duration of aggression, aggression between siblings was removed before further analysis of ALL, UNI and FIGHT networks. There was no correlation between the proportion of littermates in the pen (littermates/ total other pigs) and the likelihood of winning fights (wins/ wins + losses), Rs= 0.051, p=0.503, so siblings fights were retained in mutual fighting models directed by fight outcome and weighted by number of fights.

Degree

All degree, in-degree and out-degree were calculated using "degree" function, with the option to normalise set to false. Degree was then normalized to account for differences in pen size and familiarity by dividing degree by the number of non-littermates in the pen. For WIN networks, fights between littermates were

included, because the number of proportion of littermates in the pen did not significantly influence the likelihood of winning fights and data were normalised by dividing degree by the number of others in the pen (n-1).

Betweenness

The betweenness centrality of a node (x) is found by firstly calculating the ratio of all of the possible paths between two nodes, a and b which run through x. This ratio is calculated for every pair of nodes in the network, and the sum of these ratios is the betweenness centrality of node x. Where several nodes are equally present on the shortest path (also known in graph theory as the "geodesic") between two nodes, the betweenness value is divided between these nodes (e.g., in a network of size 5 (figure 6.2.a) where only nodes 4 and 5 are not connected, nodes 1, 2 and 3 have a betweenness centrality of 0.33).

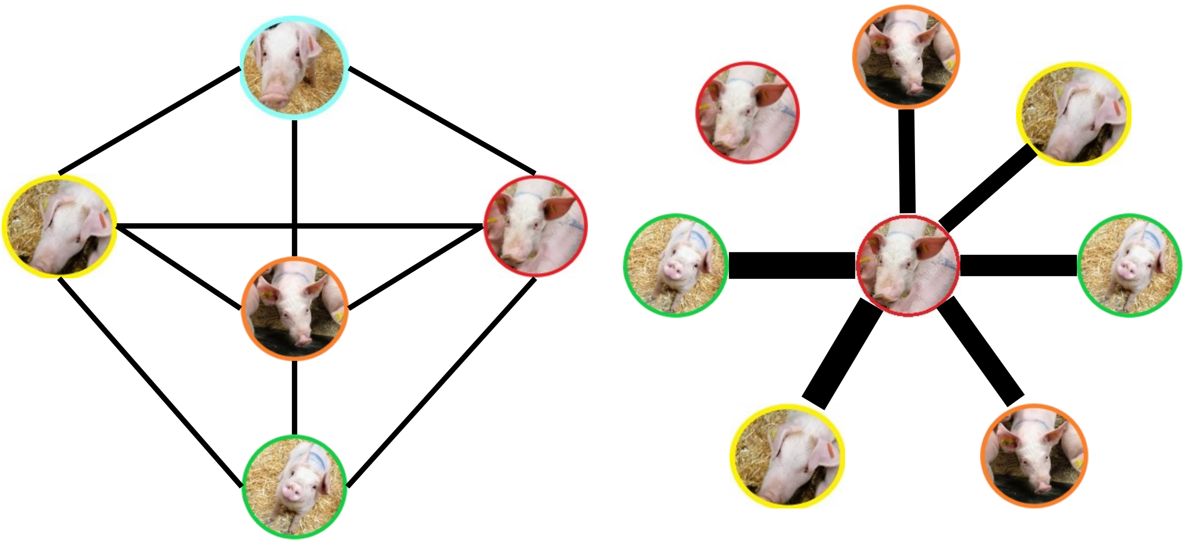

Figure S1 Model networks to demonstrate the trait of betweenness centrality. In both diagrams, nodes are labelled by unweighted betweenness. a) Model network in which pigs are represented by pig faces and edges are represented by black lines. b) Model network in which shortest paths are represented by black lines. Node outline colour indicates node litter. The pig in the centre with a red outline lies on the shortest path between two non-littermates on 12 occasions.

Unweighted betweenness centrality was calculated using the function "betweenness", with the option to normalize set to false. As edges between littermates are not included in this analysis, betweenness is centralised according to the number of non-sibling dyads which did not include the focal pig. The maximum theoretical betweenness centrality would apply if a network was completely centralised (figure S1 b) (e.g., where one pig exchanges aggression with all others but no other connections are present). I calculated this value for each pig by counting the number of dyads in the pen which were unrelated to

the focal node (x) and themselves unrelated. For example, the maximum betweenness centrality value for pigs in the smallest network (8 pigs from 4 litters, with one sibling each) is 12 (figure S1 b). For pigs in the largest network (16 pigs from 7 litters) the maximum betweenness is 80 for pigs with one sibling in the pen and 60 for pigs with 3 siblings in the pen.

A shorter path with weak ties *or* a longer path with stronger ties may be the least costly route for information (or in this case, aggression) to flow between two nodes, therefore weighted betweenness was calculated, which takes account of both the number of ties and the strength of connections (duration). While there is no theoretical maximum for weighted centrality measures, weighted betweenness was also divided by the theoretical maximum unweighted betweenness centrality per pen to correct for pen size. I avoided scaling metrics between 0 and 1 in order to be able to compare between individuals with the same characteristics (e.g. sex, body weight and cognitive ability) across pens rather than only relative to their pen-mates.

Eigenvector

Eigenvector centrality is the sum of the degree centrality of all nodes directly connected to a focal node. Eigenvector centrality was only calculated for WIN networks, and I applied the function "eigen_centrality" in igraph, specifying for the calculation to include the direction of the edges. Since eigenvector centrality may be limited by pen size, we scaled the result and compared this to the unscaled result. Factors in the null and cognition models affected scaled and unscaled eigenvector with the same degree of statistical significance (see supplementary table S3), therefore we considered the "scaled" outcome redundant and report unscaled values only, as these allow us to compare scores calculated in the same way for pigs in different pens.

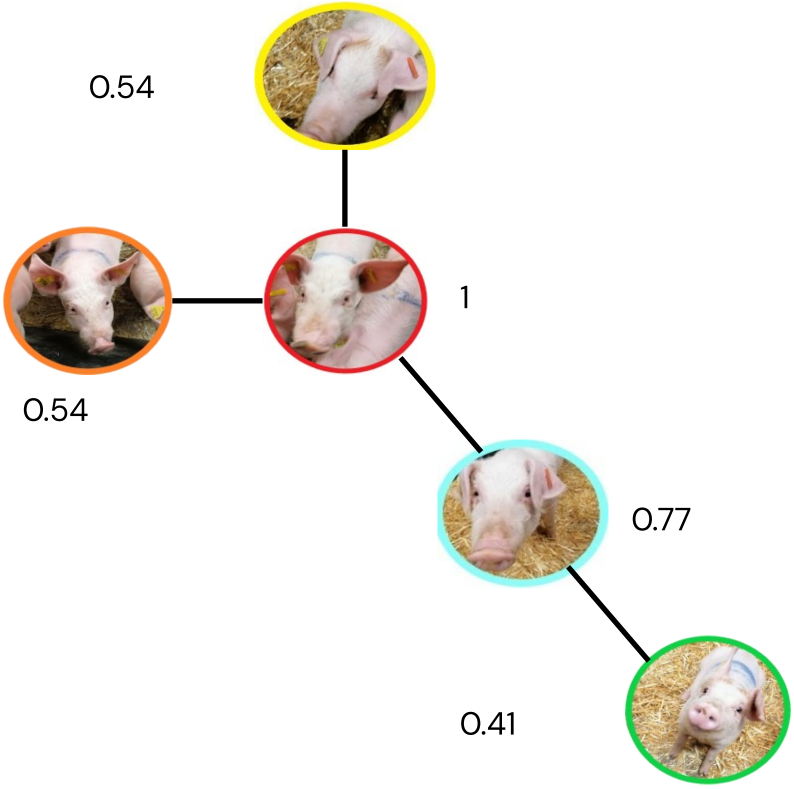

Figure S2. Model network to demonstrate the trait of eigenvector centrality. Nodes are labelled by eigenvector centrality and node size is proportional to eigenvector centrality. The pigs in yellow, orange and green circles each have one direct connection (degree=1), but the pig in green has a lower eigenvector centrality because it is connected to a pig with a degree of 2, while the pigs in yellow and orange circles are connected to a pig with a degree of 3.

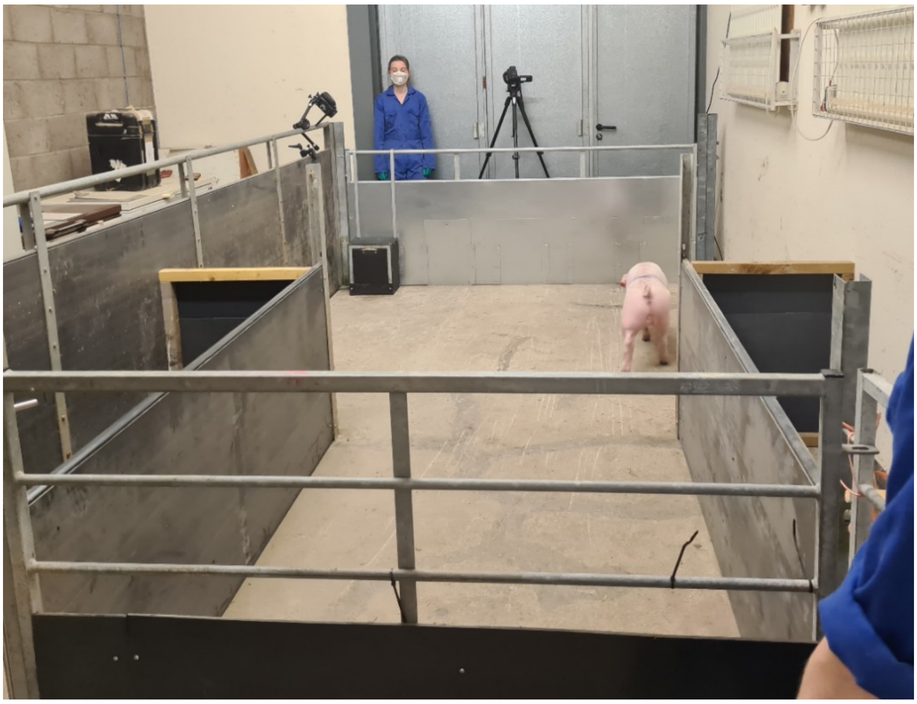

Figure S3. Diagram of the arena used for the spatial discrimination task. The box contained a food reward or an inaccessible reward as a non-rewarded stimulus to control for olfactory cues. Pigs have to push their head through the flap to reach the reward. Pigs are presented with one option per trial. The location of the positive and negative reference cues (left/right) was balanced between pigs and remained consistent for each pig.

Supplementary results

The total duration of agonistic behaviour was strongly positively skewed (Table S1) and 58% was damaging aggression (51% mutual fighting + 7% unilateral aggression). Pushing, biting and fighting all correlated moderately or strongly (table S2). Biting and bullying were both unilateral, non-reciprocated forms of aggression and correlated strongly (0.702) and were combined as "unilateral aggression".

Mounting

Mounting was excluded from the category of agonistic behaviour, although it can be an expression of dominance, performed in place of aggression[29]. Mounting was performed by a small number of pigs: 10% of pigs performed 76% of total mounts, whilst 50% of pigs performed no mounting. Mounting was mostly performed by males (88%) and recipients were most often female (58%). Mounting did not correlate with agonistic behaviour: display r=-0.06, p=0.41; non-

damaging aggression -0.018, p=0.82; pushing r=-0.075, p=0.32; bites r=0.054, p=0.47; mutual fighting -0.135, p=0.08; bullying -0.021, p=0.78. Hence mounting was excluded.

Table S1. Descriptive statistics of aggressive behaviour per pig

	**Median**	**Min**	**Max**	**Skewness**	**Kurtosis**
**Total aggression delivered (s)**	633	35	2982	1.63	4.27
**Total aggression received (s)**	602	86	2963	1.85	5.10
**Unilateral aggression delivered (s)**	45	2	519	2.72	9.98
**Unilateral aggression received (s)**	52	8	313	1.90	4.70
**Mutual fighting duration (s)**	320	5	1272	0.95	0.94
**Total conclusive fights**	12	0	30	0.48	-0.28
**Wins**	6	0	27	0.84	0.67
**Losses**	6	0	21	1.04	1.03
**Lesion counts**					
**Anterior 24h**	77	0	234	0.59	0.12
**Central 24h**	8	0	53	1.26	1.32
**Posterior 24h**	3	0	85	3.87	20.98
**Anterior 7d**	10.5	0	54	1.70	3.47
**Central 7d**	1	0	23	3.09	13.68
**Posterior 7d**	1	0	9	1.62	2.61
**Anterior 14d**	9	0	41	1.33	2.20
**Central 14d**	1	0	11	1.90	3.89
**Posterior 14d**	1	0	10	1.54	3.13

Lesion counts are included for 175 pigs at 24 hours post-mixing, 174 pigs at 7 days after mixing and 171 pigs at 14 days after mixing, because individual pigs were occasionally removed from the finisher shed and placed in a hospital pen or euthanised due to health issues

Table S2: Correlation between total duration of aggressive behaviours per individual in the initial 5 hours after regrouping

	Display	NDA	Pushing	Bites	Fight	Bullying	Mounting
Display		**0.28**	0.105	0.155	0.103	0.05	-0.063
NDA	**<0.001**		**0.419**	**0.593**	**0.397**	**0.379**	-0.018
Pushing	0.17	**<0.001**		**0.353**	**0.65**	0.154	-0.075
Bites	0.04	**<0.001**	**<0.001**		**0.456**	**0.702**	0.054
Fight	0.17	**<0.001**	**<0.001**	**<0.001**		**0.352**	-0.135
Bullying	0.52	**<0.001**	0.04	**<0.001**	**<0.001**		-0.021
Mounting	0.41	0.82	0.32	0.47	0.08	0.78	

NDA= Non-damaging aggression. Correlation coefficients are above the diagonal and p-values below.

Transformation and categorisation of outcome variables

In UNI networks, unweighted degree and outdegree were categorised as above/ below Q1 (degree: 0.86, outdegree: 0.63) and in FIGHT networks, betweenness was categorised as above/below Q3 (unweighted betweenness: 1.17, weighted

betweenness: 8.86). Central and posterior lesion counts 1 and 2 weeks after mixing were very low and skewed (table 3), so binary outcome variables (lesions present/absent) were analysed.

Table S3. Descriptive statistics of network traits.

**Network type**	**Centrality**	**Median**	**Min**	**Max**	**Skew**	**Kurtosis**	**Transformation**
ALL	Degree	1.00	0.79	1.00	-2.20	4.09	NA
	Weighted degree	109	11	585	1.84	4.40	Log(x+1)
	Indegree	1.00	0.79	1.00	-2.20	4.09	NA
	Weighted indegree	53	7	296	1.93	4.72	Log (x+1)
	Outdegree	1.00	0.79	1.00	-2.20	4.09	NA
	Weighted outdegree	57	3	298	1.72	3.97	Log (x+1)
	Betweenness	0.20	0	1.63	0.98	0.03	Log (x+1)
	Weighted betweenness	2.03	0	31.00	2.20	6.82	Log (x+1)
UNI	Degree	1.00	0.50	1.00	-1.56	2.88	Binary (logit)
	Weighted degree	11	2	60	2.07	6.32	Log (x+1)
	Indegree	0.79	0.27	1.00	-0.42	-0.34	None
	Weighted indegree	5	1	31	2.00	5.63	Log (x+1)
	Outdegree	0.82	0.08	1.00	-0.93	0.22	Binary (logit)
	Weighted outdegree	5	0	52	2.87	11.85	Log (x+1)
	Betweenness	1.12	0.04	7.82	2.05	5.79	Log (x+1)
	Weighted betweenness	1.43	0.111	9.73	1.61	2.78	Log (x+1)
FIGHT	Degree	0.67	0.15	1.00	-0.13	-0.60	None
	Weighted degree	56	0	255	1.02	0.95	√
	Betweenness	0.61	0	5.04	1.95	4.25	Binary (logit)
	Weighted	1.91	0	59.4	2.28	6.29	Binary (logit)
WIN	Indegree	0.33	0	0.88	0.33	-0.13	None
	Outdegree	0.33	0	1.00	0.38	-0.36	Log (x+1)
	Eigenvector	0.21	0	0.75	0.76	0.27	Log (x+1)

Where necessary, centrality measures were transformed so that the residuals of the linear mixed models were approximately normally distributed.

Table S4. Spearman's rank correlation (Rs) between unweighted and weighted centrality measures.

Centrality measure	Network type	Rs	P
Degree	ALL	0.293	<0.001
	UNI	0.286	<0.001

	FIGHT	0.518	<0.001
Indegree	ALL	0.279	<0.001
	UNI	-0.055	0.474
	WIN	**0.864**	**<0.001**
Outdegree	ALL	0.297	<0.001
	UNI	0.711	<0.001
	WIN	0.916	<0.001
Betweenness	ALL	0.141	0.063
	UNI	**0.824**	**<0.001**
	FIGHT	0.505	<0.001

Values in bold are strongly correlated, Rs>0.8.

Table S5. Effect of inclusion in the spatial discrimination task on social network centrality

		**Included in spatial discrimination task**		
**Network**	**Centrality trait**	Yes	No	F/ X2 (p)
**ALL**	**Weighted degree**	113 (103- 125)	104 (92- 117)	0.74 (0.390)
	**Weighted indegree**	55 (48- 62)	55 (50 – 61)	0.02 (0.881)
	**Weighted outdegree**	54 (49- 60)	50 (44- 57)	0.45 (0.503)
	**Betweenness**	0.64 (0.60- 0.68)	0.71 (0.66- 0.77)	2.74 (0.098)
	**Weighted betweenness**	2.42 (2.144- 2.723)	2.42 (2.04- 2.85)	0 (0.983)
**UNI**	**Degree†**	0.80 (0.76- 0.84)	0.75 (0.68- 0.80)	-0.61 (0.436)
	**Outdegree†**	0.77 (0.73- 0.80)	0.77 90.70- 0.83)	0.00 (0.96)
	**Indegree**	0.77 (0.75- 0.78)	0.74 (0.72- 0.77)	0.67 (0.414)
	**Weighted degree**	11 (9-12)	12 (10-13)	0.80 (0.371)
	**Weighted outdegree**	4.0 (3.6- 4.5)	3.8 (3.2- 4.5)	0.037 (0.847)
	**Weighted indegree**	4.95 (4.60-5.33)	5.05 (4.53- 5.64)	0.02 (0.888)
	**Betweenness**	1.14 (1.07- 1.22)	1.21 (1.09- 1.33)	0.20 (0.655)
	**Weighted betweenness**	1.48 (1.37- 1.59)	1.64 (1.46- 1.83)	0.59 (0.441)
**FIGHT**	**Degree**	0.64 (0.61- 0.66)	0.68 (0.64- 0.71)	1.45 (0.229)
	**Weighted degree**	64 (59- 70)	57 (50- 64)	1.02 (0.312)
	**Betweenness†**	2.15	1.85	0.826 (0.363)
	**Weighted betweenness†**	0.73	0.99	0.59 (0.442)
**WIN**	**Indegree**	0.36 (0.34- 0.37)	0.37 (0.34- 0.39)	0.54 (0.463)
	**Outdegree**	0.035 (0.32- 0.37)	0.30 (0.31- 0.38)	0 (0)
	**Weighted indegree**	0.52 (0.49- 0.55)	0.48 (0.43- 0.52)	0.56 (0.454)
	**Weighted outdegree**	0.51 (0.46- 0.55)	0.46 (0.40- 0.52)	0.62 (0.430)
	**Eigenvector (unscaled)**	0.21 (0.20- 0.22)	0.25 (0.23- 0.27)	2.46 (0.119)
	**Eigenvector (scaled)**	0.48 (0.45- 0.66)	0.50 (0.46- 0.70)	0.14 (0.709)

† Results were derived from GLMs, log-it values are transformed to odds ratios (OR) and confidence intervals are estimated using e^(OR+/- s.e.).

Table S6. Effect of inclusion in the reversal learning test on social network centrality

		Included in reversal learning test		
**Network**	**Centrality trait**	Yes	No	F/ X^2 (p)
**ALL**	**Weighted degree**	113 (103- 125)	106 (94- 118)	0.34 (0.558)
	**Weighted indegree**	53 (47-58)	59 (52- 66)	1.02 (0.312)
	**Weighted outdegree**	55 (49- 61)	51 (46- 58)	0.207 (0.649)
	**Betweenness**	0.644 (0.598- 0.690)	0.672 (0.621- 0.724)	0.994 (0.319)
	**Weighted betweenness**	2.29 (1.99- 2.61)	2.63 (2.27- 3.04)	0.660 (0.417)
**UNI**	**Degree†**	0.71 (0.70- 0.72)	0.72 (0.71- 0.74)	0 (1)
	**Indegree**	0.77 (0.73- 0.80)	0.74 (0.69- 0.79)	0.69 (0.407)
	**Outdegree†**	0.82 (0.78- 0.85)	0.70 (0.64- 0.75)	-3.47 (0.062)
	**Weighted degree**	11 (10-12)	11 (10- 12)	0.21 (0.644)
	**Weighted outdegree**	4.2 (3.7- 4.7)	3.6 (3.1- 4.2)	0.69 (0.408)
	**Weighted indegree**	5.10 (4.70- 5.55)	4.85 (4.41- 5.35)	0.22 (0.636)
	**Betweenness**	1.18 (1.09- 1.26)	1.14 (1.04- 1.24)	0.07 (0.797)
	**Weighted betweenness**	1.45 (1.34- 1.58)	1.62 (1.47- 1.78)	0.82 (0.366)
**FIGHT**	**Degree**	0.65 (0.61- 0.66)	0.64 (0.62- 0.68)	0.009 (0.926)
	**Weighted degree**	66 (60-72)	57 (58- 73)	1.67 (0.20)
	**Betweenness†**	2.08	2.03	0.028 (0.868)
	**Weighted betweenness†**	0.87	0.93	0.28 (0.596)
**WIN**	**Indegree**	0.36 (0.35- 0.38)	0.36 (0.34- 0.38)	0.58 (0.446)
	**Outdegree**	0.35 (0.32-0.37)	0.34 (0.31-0.37)	0 (0)
	**Weighted indegree**	0.51 (0.48- 0.54)	0.50 (0.46- 0.54)	0.02 (0.895)
	**Weighted outdegree**	0.52 (0.47- 0.57)	0.46 (0.40- 0.51)	0.81 (0.367)
	**Eigenvector (unscaled)**	0.20 (0.18- 0.21)	0.25 (0.23- 0.27)	5.22 (0.021)
	**Eigenvector (scaled)**	0.49 (0.46- 0.70)	0.48 (0.44- 0.66)	0.06 (0.811)

† Results were derived from GLMs, log-it values are transformed to odds ratios (OR) and confidence intervals are estimated using e^(OR+/- s.e).

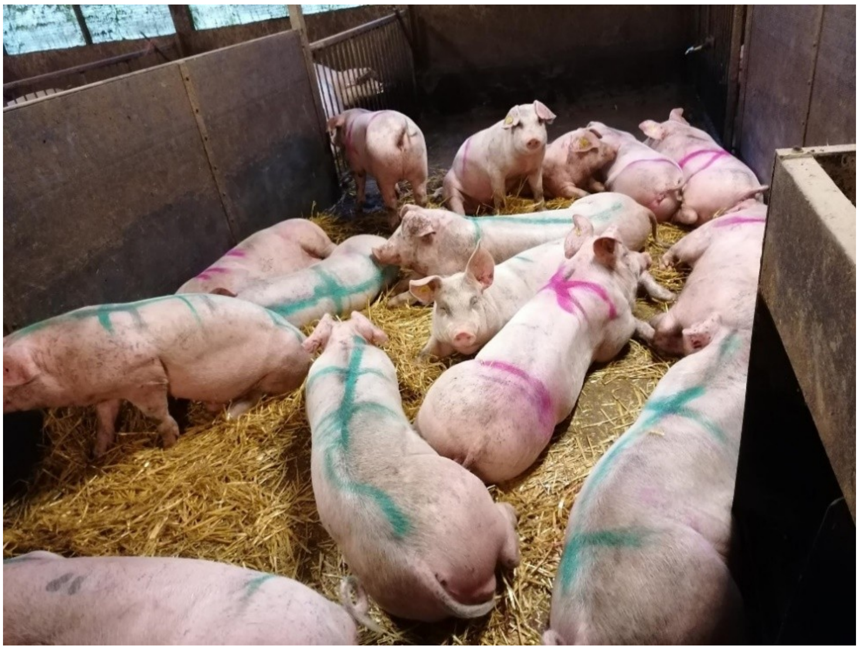

Figure S4. Photograph to illustrate high stocking density in finisher pens which may prevent pigs from escaping aggression regardless of cognitive ability.
