# Supplementary figures and images for: Cognitive and non-cognitive factors predict pigs’ positions in an aggression social network
Source: Sci Rep. 2025 May 20;15:17439. doi: 10.1038/s41598-025-02023-w (PMC12092641; doi:10.1038/s41598-025-02023-w)

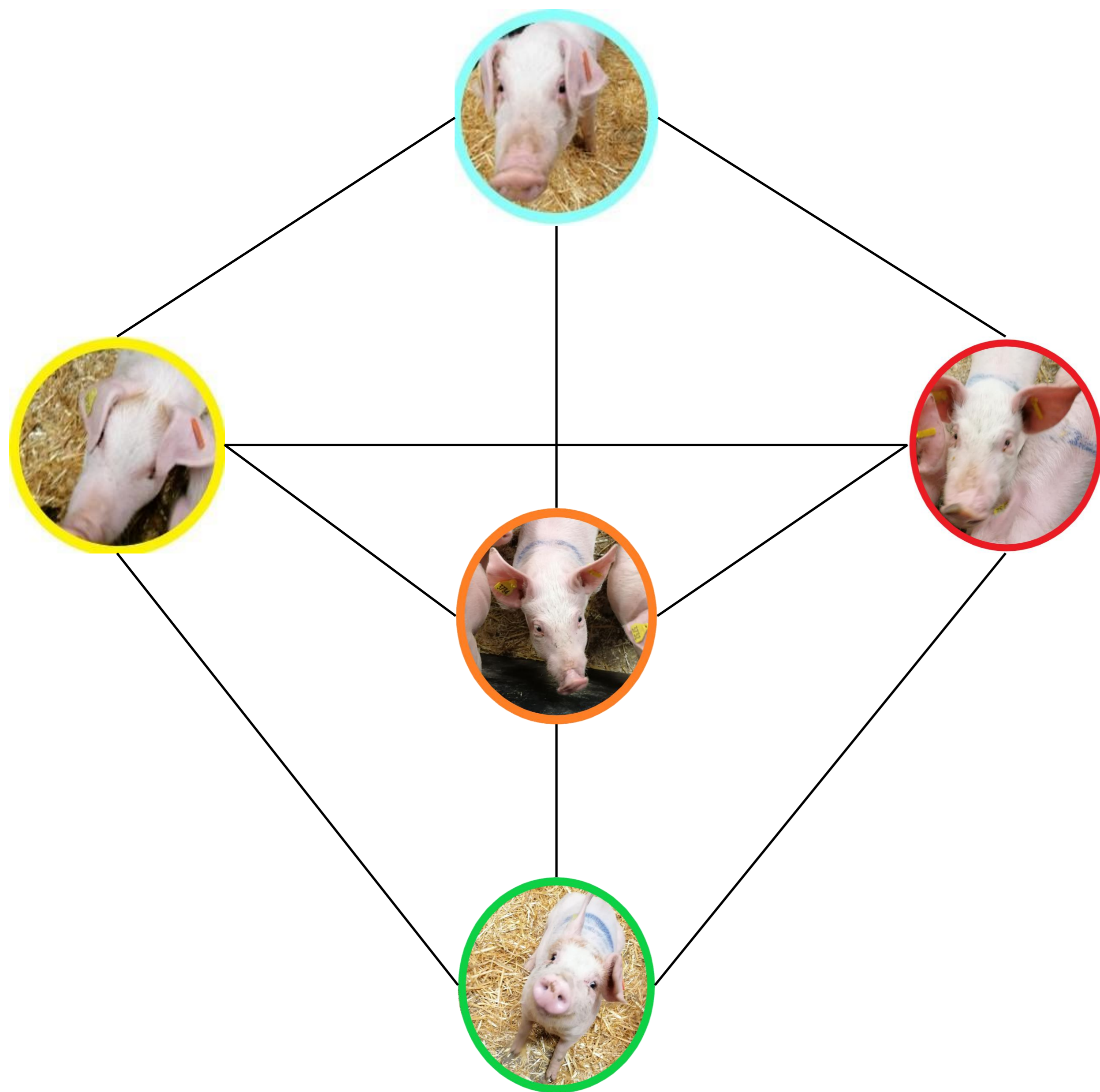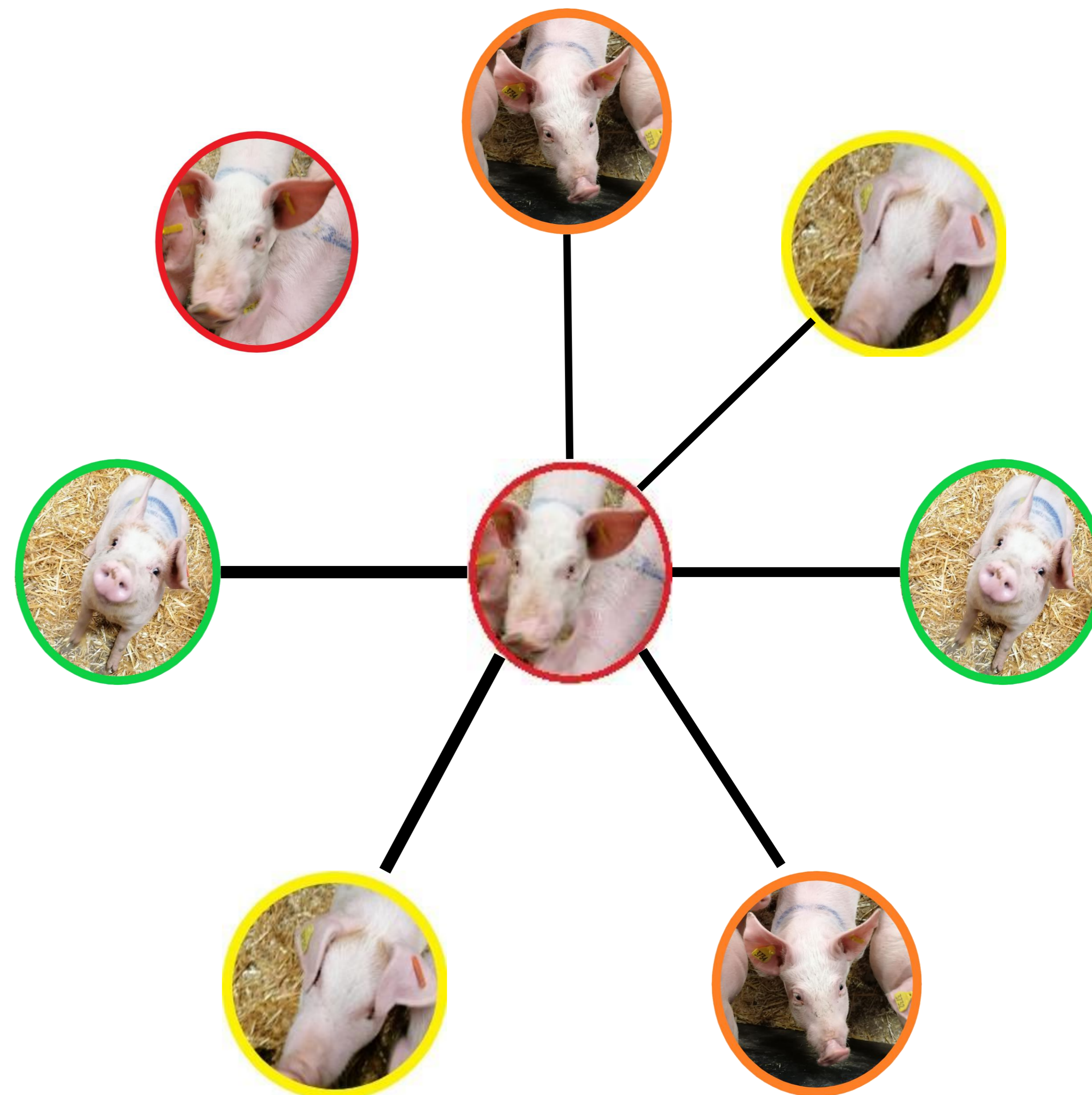

Supplement: Supplementary file 2 — Supplementary information 2. [file 41598_2025_2023_MOESM2_ESM.pdf]

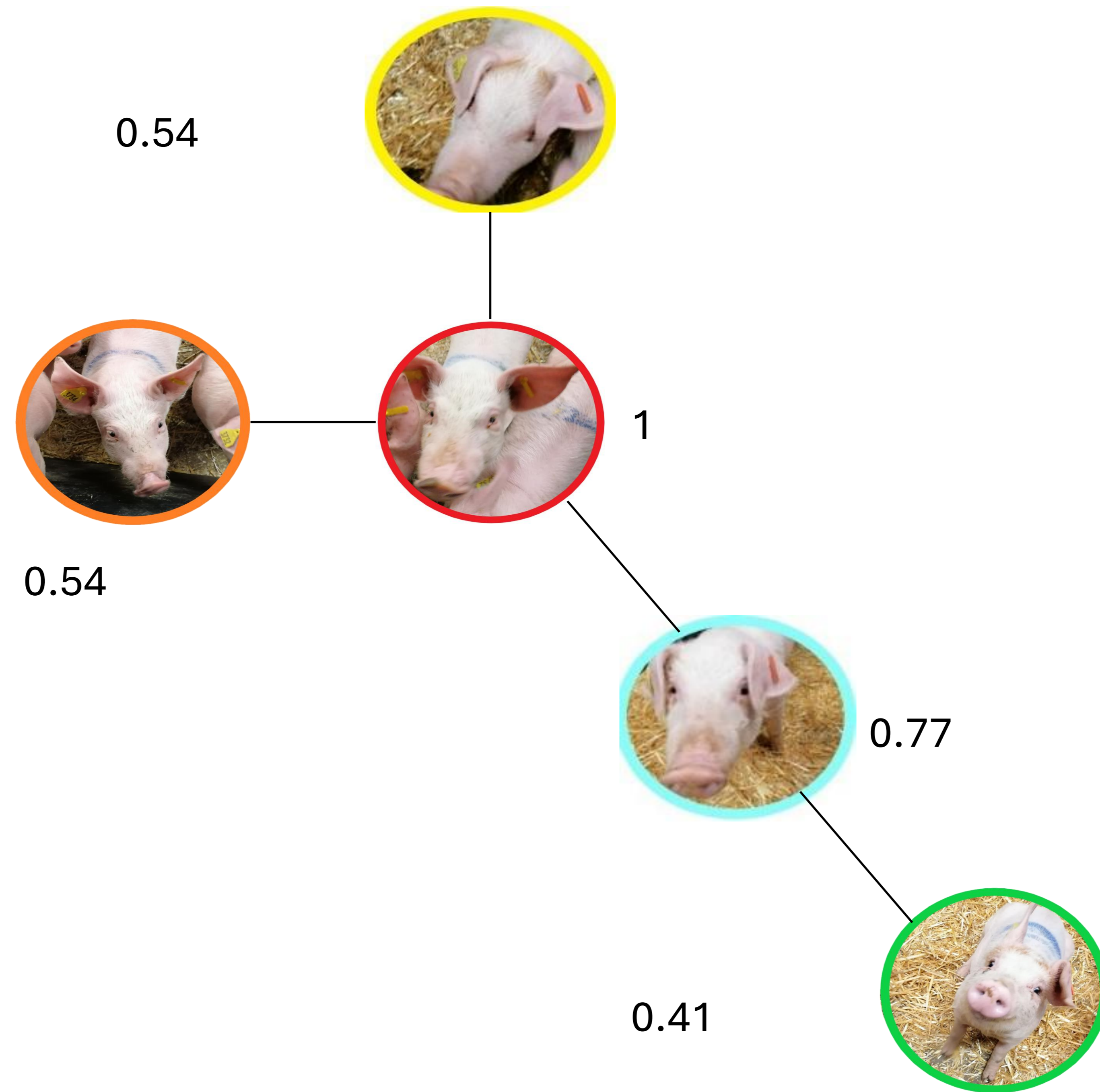

Supplement: Supplementary file 3 — Supplementary information 3. [file 41598_2025_2023_MOESM3_ESM.pdf]

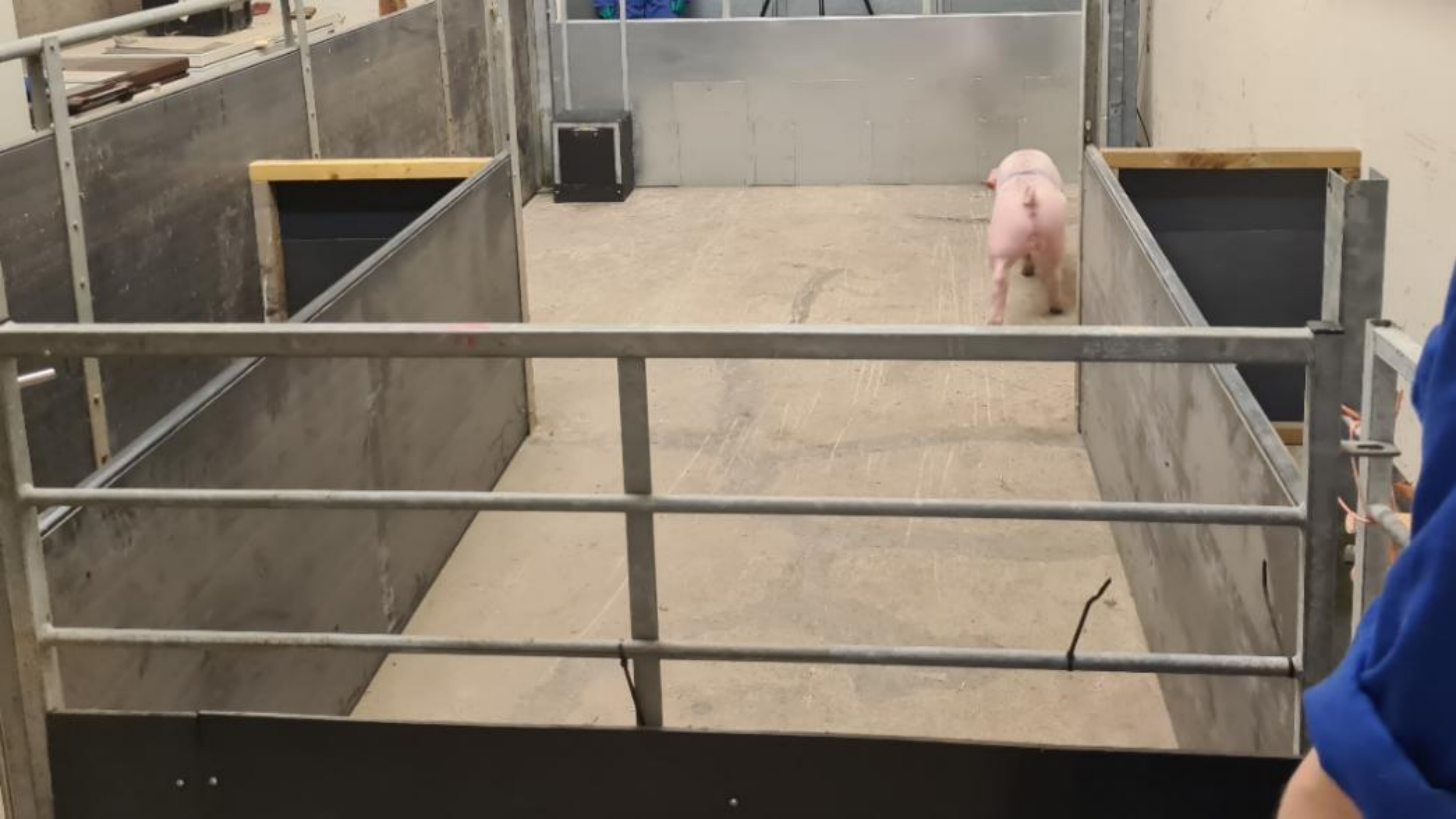

Supplement: Supplementary file 4 — Supplementary information 4. [file 41598_2025_2023_MOESM4_ESM.pdf]

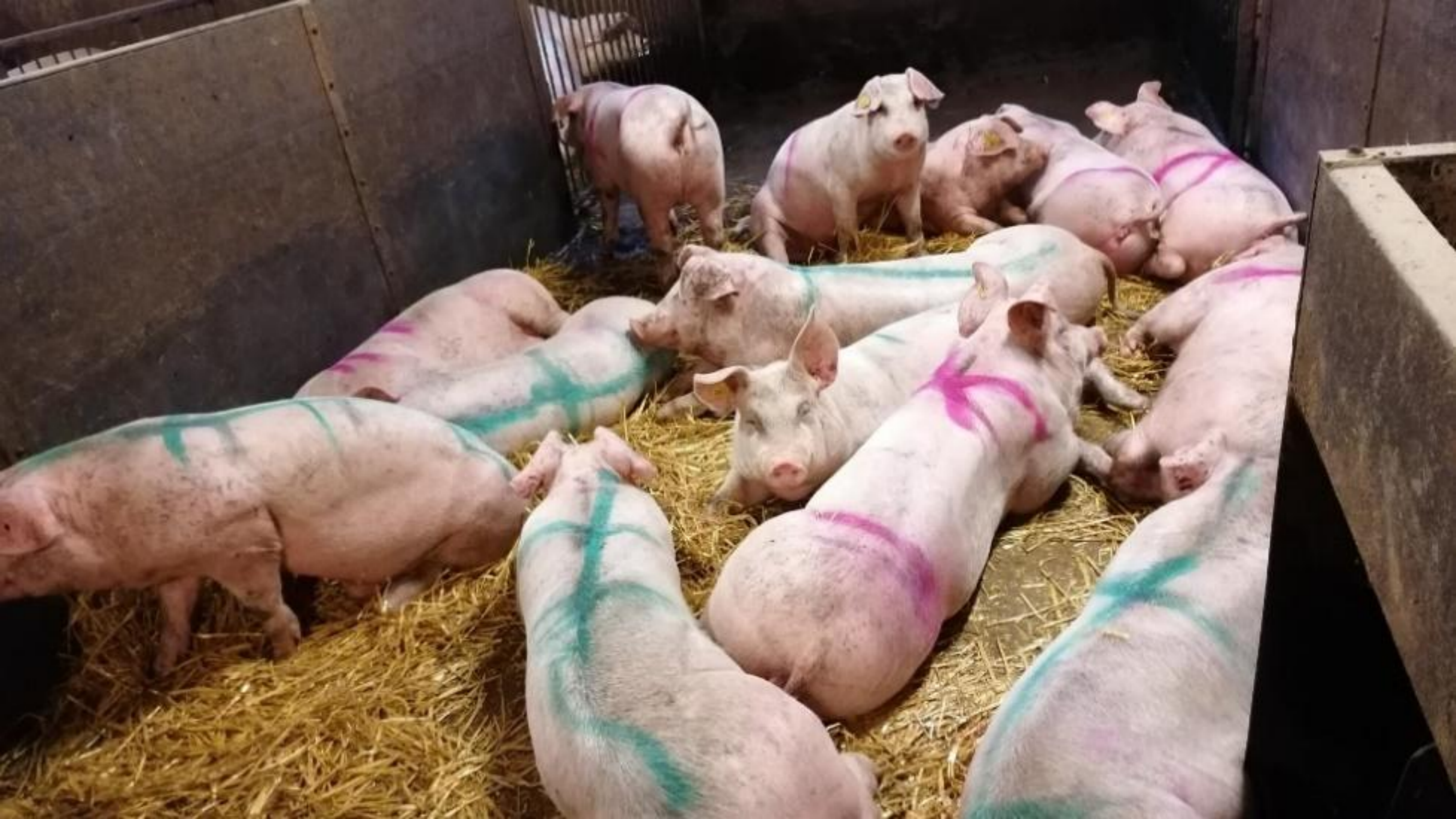

Supplement: Supplementary file 5 — Supplementary information 5. [file 41598_2025_2023_MOESM5_ESM.pdf]
